# Supplementary material for: Group testing via hypergraph factorization applied to COVID-19
Source: Nat Commun. 2022 Apr 5;13:1837. doi: 10.1038/s41467-022-29389-z (PMC8983763; doi:10.1038/s41467-022-29389-z)
Supplement: Supplementary file 1 — Supplementary Information [file 41467_2022_29389_MOESM1_ESM.pdf]

SUPPLEMENTARY MATERIAL
"Group testing via hypergraph factorization applied to COVID-19"

David Hong[1] Rounak Dey[2] Xihong Lin[2,3,4] Brian Cleary[4] Edgar Dobriban[1]

[1]Department of Statistics and Data Science, The Wharton School, University of Pennsylvania, Philadelphia, PA, USA

[2]Department of Biostatistics, Harvard T.H. Chan School of Public Health, Boston, MA, USA

[3]Department of Statistics, Harvard University, Cambridge, MA, USA

[4]Broad Institute of MIT and Harvard, Cambridge, MA 02142 USA

List of Supplementary Notes

S1 Maximally balanced designs **S3**

S2 On the nontriviality of developing maximally balanced designs **S4**

S3 Maximal balance and hypergraph factorization **S6**

S4 Efficient constructions of hypergraph factorizations **S8**

S4.1 Hypergraph factorization of the complete hypergraph for $q = 1$ . . . S8

S4.2 Hypergraph factorization of the complete hypergraph for $q = 2$ . . . S8

S4.3 Hypergraph factorization of the complete hypergraph for $q = 3$ . . . S9

S5 Relationship to design theory **S11**

S6 Reordering HYPER pool designs to aid lab implementation **S12**

S7 Correcting for false negatives in HYPER **S13**

S8 Balanced array designs **S14**

S9 Reed-Solomon Kautz-Singleton code-based designs **S16**

S10 Performance characterization under a common statistical model **S19**

S10.1 An upper bound on the expected number of tests in the noiseless case . . . S19

S10.2 Optimal efficiency for HYPER with $q = 2$ . . . S21

S10.3 Generalization to the noisy case . . . S22

List of Supplementary Figures

1 Performance under a common statistical model . . . S26

2 Comparison of two design problems and their bounds . . . S27

3 Comparison of plate-based array designs with additional HYPER designs . . . S28

4 Comparison of P-BEST with additional HYPER designs . . . S28

5 Comparison with additional designs . . . S29

6 Comparison with balanced array and code-based designs with matching parameters . . . S30

7 Impact of imbalanced designs . . . S31

8 Efficiency and sensitivity of pooled testing during a simulated epidemic (Fig. 2) repeated for a 25-fold reduction in the limit of detection (from 100 to 4) . . . S32

9 Efficiency and sensitivity of pooled testing during a simulated epidemic (Fig. 2) repeated with a two-wave epidemic . . . S33

10 Detailed comparison corresponding to Fig. 3 . . . S34

11 Comparison of pooling methods under resource constraints (Fig. 3 and Supplementary Fig. 10) repeated for day 53 (prevalence of 0.1%) . . . S35

12 Comparison of pooling methods under resource constraints (Fig. 3 and Supplementary Fig. 10) repeated for day 80 (prevalence of 1.06%) . . . S36

13 Comparison of pooling methods under resource constraints (Fig. 3 and Supplementary Fig. 10) repeated for day 83 (prevalence of 1.36%) . . . S37

14 Comparison of pooling methods under resource constraints (Fig. 3 and Supplementary Fig. 10) repeated for day 84 (prevalence of 1.48%) . . . S38

15 Comparison of pooling methods under resource constraints (Fig. 3 and Supplementary Fig. 10) repeated for day 90 (prevalence of 2.46%) . . . S39

16 Comparison of pooling methods under resource constraints (Fig. 3 and Supplementary Fig. 10) repeated for day 93 (prevalence of 3.15%) . . . S40

17 Comparison with balanced array designs under resource constraints for day 53 (prevalence of 0.1%) . . . S41

18 Comparison with balanced array designs under resource constraints for day 80 (prevalence of 1.06%) . . . S42

19 Comparison with Reed-Solomon Kautz-Singleton (RS-KS) code-based designs under resource constraints for day 53 (prevalence of 0.1%) . . . S43

20 Comparison with Reed-Solomon Kautz-Singleton (RS-KS) code-based designs under resource constraints for day 80 (prevalence of 1.06%) . . . S44

21 Definite defective (DD) decoding . . . S45

S1 Maximally balanced designs

We seek designs that are maximally balanced in three ways. Here we illustrate each way through some examples:

1. Assign all individuals to the same number q of pools

Imbalanced design

Individual	1	2	3	4	5	6
Pool	AB	C	DE	F	AB	CD

Balanced design

Individual	1	2	3	4	5	6
Pool	AB	CD	EF	BC	DF	AE

In the imbalanced design, all individuals are in two pools except individuals 2 and 4, who are in one pool. The balanced design assigns all individuals to two pools.

2. Assign the m pools as evenly as possible

Imbalanced design

Individual	1	2	3	4	5	6
Pool	AB	AC	AD	AE	AF	BC

Balanced design

Individual	1	2	3	4	5	6
Pool	AB	CD	EF	BC	DF	AE

In the imbalanced design (lexicographic pooling), pool A has five individuals, pools B and C have two, and pools D-F have only one. All pools in the balanced design have two individuals.

3. Assign the $\binom{m}{q}$ possible pool combinations as evenly as possible

Imbalanced design

Individual	1	2	3	4	5	6
Pool	AB	CD	EF	AB	CD	EF

Balanced design

Individual	1	2	3	4	5	6
Pool	AB	CD	EF	BC	DF	AE

In the imbalanced design (consecutive pooling), pool combinations AB, CD and EF are each used twice but none of the other combinations (e.g., AC) are used.

Formally, maximally balanced designs can be defined as follows.

Definition 1: Let $P \in \{0,1\}^{m\times n}$ denote a pooling design with n individuals and m pools, where $P_{ij} = 1$ means that pool i contains individual j and $P_{ij} = 0$ otherwise. We say P is maximally balanced if for some q

$$\text{i)}\ q_j = q \quad \text{for all } j,$$

$$\text{ii)}\ \max_{i=1,\ldots,m} k_i - \min_{i=1,\ldots,m} k_i = \begin{cases} 0 & \text{if } n \text{ is a multiple of } m/q, \\ 1 & \text{otherwise,} \end{cases}$$

$$\text{iii)}\ \max_{\pi\in\Pi_q} \sigma_\pi - \min_{\pi\in\Pi_q} \sigma_\pi = \begin{cases} 0 & \text{if } n \text{ is a multiple of } \binom{m}{q}, \\ 1 & \text{otherwise,} \end{cases}$$

where $q_j := \sum_{i=1}^m P_{ij}$ is the number of pools individual j is assigned to, $k_i := \sum_{j=1}^n P_{ij}$ is the size of pool i, σ_π is the number of individuals assigned to the pool combination π, and $\Pi_q := \{\pi \subseteq \{1,\ldots,m\} : |\pi| = q\}$ is the set of all possible combinations of q pools chosen from $\{1,\ldots,m\}$.

We say that maximally balanced pools are also perfectly balanced when they are assigned exactly the same number of times, i.e., not just as evenly as possible. Likewise, we say that the pool assignments are perfectly balanced when they are assigned exactly the same number of times.

S2 On the nontriviality of developing maximally balanced designs

For $q = 1$, which corresponds to classical Dorfman testing, each individual needs to be assigned to a single pool. Balance is easily achieved here by cycling through the pools until all individuals are assigned. For example, to assign $n = 8$ individuals to $m = 6$ pools (A-F), assign individual 1 to pool A, individual 2 to B, and so on, yielding the following assignments:

Individual	1	2	3	4	5	6	7	8
Pool	A	B	C	D	E	F	A	B

All the $n = 8$ individuals are assigned to $q = 1$ pool, the $m = 6$ pools are assigned as evenly as possible (A and B are assigned twice and the rest are assigned once), and pool combinations are just the pools themselves when $q = 1$.

However, achieving maximal balance for $q > 1$ turns out to be highly nontrivial in general. Consider a straightforward approach of listing the pools and assigning individuals to consecutive pairs (e.g., AB, CD, EF, AB, ...). This design produces balanced pools but under-utilizes the combinatorial space (e.g., it does not use AC) and is inefficient. Alternatively, one might consider cycling through all possible pool combinations in their natural lexicographic order (i.e., AB, AC, AD, AE, ...). This design produces balanced pool combinations but now has imbalanced pools, which dilute the individuals unevenly and place some at a higher risk of false negatives than others. For instance, using all pairs in lexicographic order for $n = 8$ individuals assigned to $q = 2$ of $m = 6$ pools, yields

Individual	1	2	3	4	5	6	7	8
Pools	AB	AC	AD	AE	AF	BC	BD	BE

which assigns A five times and F only once. As a result, this design is not uniformly sensitive across individuals. For example, individual 1 undergoes a five-fold dilution in pool A and a four-fold dilution in pool B, while individual 5 is diluted in pool A but not at all in pool F. Uneven dilution of viral loads can lead to differential false negative test results, leaving individual 1 with a higher risk of false negatives than individual 5. Clearly, alternative orderings could be more balanced, and the challenge is how to systematically identify these.

One approach is to generate random designs such as random assignment[1], which assigns each individual to q of m pools chosen uniformly at random. This design treats both pools and pool combinations uniformly on average, but any given random draw may assign them very unevenly in practice, especially when m, n, and q are not very large. For the example above, this approach draws one of the $\binom{m}{q}^n = 15^8$ possible designs uniformly at random, so pool sizes (individuals per pool) can range all the way from zero to n. Likewise for pool combinations. In fact, 98.7% of the random draws in this case will not be maximally balanced, i.e., the pools or pool combinations are not assigned as evenly as possible, as can be verified by exhaustive search. Hence, it is rare for these randomly generated designs to both treat individuals uniformly and maximally use all available pool combinations. With high probability, the random assignment design does not achieve our goal.

One could also consider searching among many random draws for a suitable design. In the small example above, it would take 180 draws to have a 90% chance of drawing at least one maximally balanced design. One might try selecting

then modifying the most balanced (in some appropriate sense) from among the many draws, but such an approach is ad-hoc, involves manual tweaking, and may still not be maximally balanced. Double-pooling [2] is an alternative random design that instead partitions the n individuals into $m/2$ pools two times. This design produces balanced pools but does not guarantee balanced pool combinations, so again involves the possibility of manual tweaking to achieve maximal balance.

Exhaustive search, on the other hand, is a systematic approach but is only practical for very small cases. The $\binom{m}{q}^n$ many designs to search through rapidly becomes intractable in general. Moreover, one must repeat the process for any change in the numbers of individuals n, number of splits q, or number of pools m; the design is not easily adapted from one environment to another. One could attempt to precompute and store designs in advance, but this then limits us to the set of designs anticipated to be useful, again reducing flexibility.

Code-based designs (like P-BEST [3]) and array designs (like plate-based arrays [4]) can be maximally balanced but may only apply or remain balanced for certain choices of n and m. For example, array designs do not use all available pool combinations so do not have maximally balanced pool combinations once the number of individuals exceeds the number of cells in the array. Code-based designs can also require significant technical expertise to adapt. These aspects all limit the flexibility and adaptability of these approaches.

As a result, though it is quite natural to desire maximally balanced designs, the problem of easily generating them is highly nontrivial. In fact, it is not initially clear that designs maximally balanced in all three ways even exist in general, let alone, an efficient way to find them. In this paper, we have shown how certain deep results from combinatorics, such as Baranyai's theorem [5] imply that such designs do in fact exist, under the minimal required conditions. Moreover, we demonstrate how to generate them under mild conditions using the combinatorics of hypergraph factorizations. For $q = 3$, we leverage a non-trivial number-theoretic construction due to Beth [6;7]. These tools enable us to develop a simple, flexible and efficient pooled testing strategy with maximally balanced pool designs.

S3 Maximal balance and hypergraph factorization

As discussed in the main article, HYPER pooling designs are guaranteed to be maximally balanced in general as a consequence of the properties of hypergraph factorization. In particular, the properties of hypergraph factorization imply that the resulting sequence of pool assignments (obtained in the setup stage) produces maximal balance for any number of individuals. Indeed, as we will now explain, this is why HYPER uses hypergraph factorization for generating the pools.

The key idea is to order the $\binom{m}{q}$ pool combinations so that each consecutive block of m/q combinations contains each pool exactly once (we require that q divides m). In our earlier example (Fig. 1), the first block of $m/q = 3$ pairs (AB, CD, EF) contains each of the pools (A-F) exactly once, as does the next block (BC, DF, AE), and so on. This property keeps pool sizes maximally balanced as individuals one-by-one get assigned to the pool pairs, because it guarantees that each pool is assigned once before any are assigned again. Likewise, the fact that each pair appears exactly once keeps the pool combinations maximally balanced. As a result, in Fig. 1, each pool was assigned four times and each pool pair was assigned once or never, making the pools perfectly balanced and the pool pairs maximally balanced. For $n = 11$ individuals, the pools would no longer be perfectly even, since pools A and D would have only 3 individuals, but they would still be maximally balanced. The same holds for any number of individuals.

Our task, therefore, is to divide the $\binom{m}{q}$ possible pool combinations into subsets, where each pool appears exactly once in each subset. This corresponds exactly to the mathematical problem of hypergraph factorization. As described in the main article and illustrated in Fig. 1, we think of the m pools as vertices and the $\binom{m}{q}$ possible pool combination as hyperedges that each connect q of the m vertices (i.e., the blue lines in Fig. 1). The resulting hypergraph (i.e., the vertices and hyperedges taken together) contains all $\binom{m}{q}$ possible hyperedges and is known as the complete hypergraph of order q on m vertices, which we denote K_m^q. The hypergraph in Fig. 1 is K_6^2. Shown in more detail, K_6^2 has the following hyperedges:

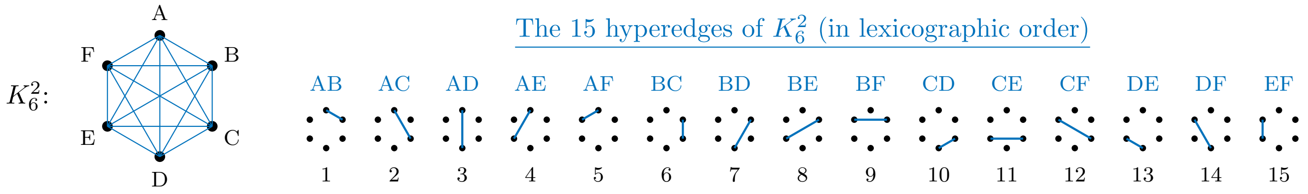

Drawing the corresponding complete hypergraph for $q = 3$ is harder, but one can quickly visualize the hyperedges as triangles connecting $q = 3$ vertices:

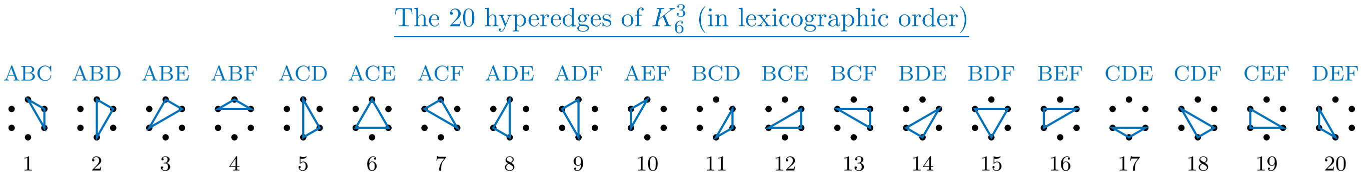

Now, recall that our task is to divide the pool combinations (i.e., hyperedges) into subsets, where each subset uses all the pools (i.e., vertices) exactly once. This problem is known in combinatorics as hypergraph factorization, and each subset is called a 1-factor of the hypergraph. Some example 1-factors for $q = 2$ and $q = 3$ are:

Two example 1-factors of K_6^2 Two example 1-factors of K_6^3

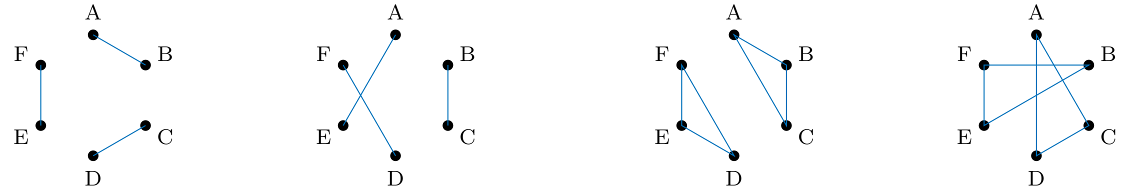

1. AB, CD, EF   2. BC, DF, AE   1. ABC, DEF   2. BEF, ACD

So, restated in these terms, our task is to factorize the complete hypergraph K_m^q into disjoint 1-factors.

In Fig. 1, the sequence of pool assignments was obtained by factorizing K_6^2 into the five disjoint 1-factors shown as little hypergraphs inside the circle of pool assignments. The first 1-factor contained hyperedges AB, CD, and EF, which gave the first $m/q = 3$ pool assignments. The next 1-factor (BC, DF, AE) gave the next $m/q = 3$ pool assignments, and so on.

Hypergraph factorization has been the subject of intense study[8;9] and has even been used for group testing[10] (though in a different way). A particularly important theorem is that such a factorization always exists (as long as q divides m), which is a deep and celebrated result from combinatorics known as Baranyai's theorem[5]. However, finding a factorization can be an incredibly challenging combinatorial problem for $q > 1$. Fortunately, efficient algorithms for constructing these factorizations have been discovered[6;7;11] for $q = 2$ (as long as m is even) as well as $q = 3$ (as long as m is a multiple of 6 and $m - 1$ is a prime number). We describe these constructions in the following sections, and we discuss how HYPER fits into the broader context of design theory in the Supplementary Material.

As mentioned in the main article, these constructions cover a very wide range of useful m and q, and we have developed an online tool (`http://hyper.covid19-analysis.org`) that implements them and generates pool assignments.

S4 Efficient constructions of hypergraph factorizations

S4.1 Hypergraph factorization of the complete hypergraph for $q = 1$

For any m, the complete hypergraph K_m^1 of order $q = 1$ is itself a 1-factor. As a result, its factorization contains only a single 1-factor: itself. So, the sequence of pool assignments is simply the pools listed (in any order). Notably, using this sequence to assign individuals to pools, reproduces Dorfman pooling as a special case.

S4.2 Hypergraph factorization of the complete hypergraph for $q = 2$

For m even, we use the following efficient method for constructing a factorization of K_m^2. Here we follow the description in Section VII-5.5 of Colbourn and Dinitz[12] and illustrate it using $m = 6$ as an example; see also page 595 of Beth et al.[8]. The construction begins with the following starter:

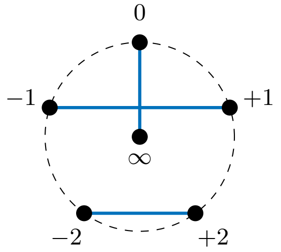

Namely, vertices $V = \{-u, \ldots, +u, \infty\}$ where $u = m/2 - 1$, i.e., $\mathbb{Z}_{m-1} \cup \{\infty\}$, with the starter 1-factor formed by edges $\{(0, \infty), (-1, +1), \ldots, (-u, +u)\}$. The remaining 1-factors are then generated by rotating the diagram as follows:

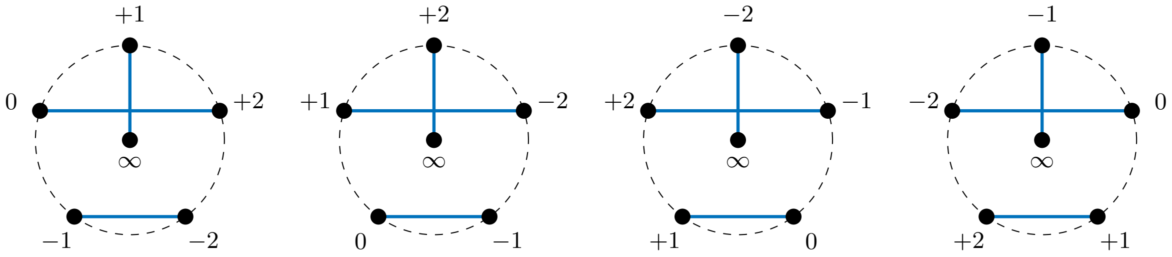

This yields $m - 1$ many 1-factors in total, that taken together form the desired hypergraph factorization of K_m^2. To connect this back to the pools, simply relabel the vertices V using the pool names. For example, using

A : 0 B : ∞ C : +1 D : −1 E : +2 F : −2

for the diagrams above yields the following 1-factors:

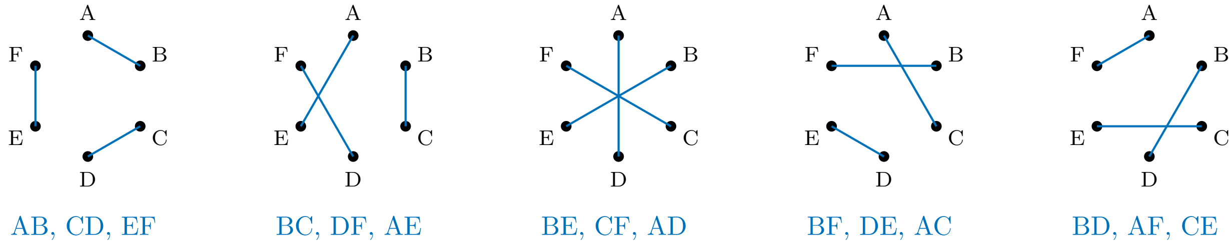

S4.3 Hypergraph factorization of the complete hypergraph for $q = 3$

For $q = 3$, we leverage a nontrivial number-theoretic construction due to Thomas Beth[6;7], which is guaranteed to work when $m = 6k$ for some integer k (i.e., the total number of pools is divisible by six) and $r = 6k - 1$ is a prime number (i.e., r is not divisible by any other number between 1 and r). Note that many designs of practical interest enjoy these properties: e.g., for pool sizes of $m = 6, 12, 24, 48$, each are divisible by six, and further we have that $r = 5, 11, 23, 47$ are prime numbers.

Algebraic background. We follow the description in Beth[6] (which appears to be difficult to access online; the construction is also presented in the thesis[7], Section 3.1, and also referenced in Tamm[11]). The construction works as follows. Consider the finite field (or Galois field) of the prime order r, $\text{GF}(r) = \mathbb{Z}/r\mathbb{Z} = F_r$. This is defined as the set of numbers $\{0, 1, \ldots, r-1\}$, with addition, multiplication, and division by nonzero elements all defined modulo r (i.e., the result is always the residue after dividing by r). To this field, we append the symbol ∞, as the result of division by zero, so that $1/\infty = 0$. We also define $c + \infty = c \cdot \infty = \infty$ for all $c \neq 0 \in F_r$. This constitutes the so-called projective line $\text{PG}(1, r)$, with the point ∞ at infinity.

Beth's construction. Now, Beth considers the fractional linear map $\pi : \text{PG}(1, r) \to \text{PG}(1, r)$ given by $\pi(x) = -(1 + x)/x$. Here, 1 denotes the additive unit of the field, while addition and division are taken modulo r. A key observation is that π is a fixed-point-free map of order three; that is, it maps $x \to \pi(x) \to \pi \circ \pi(x) \to \pi \circ \pi \circ \pi(x) = x$, such that all intermediate values are distinct. Thus, these orbits of π are sets of size three that partition $\text{PG}(1, r)$. Let $O = \{A_i, i = 1, \ldots, (r+1)/3\}$ be the partition of $\text{PG}(1, r)$ into orbits (and note that the size of $\text{PG}(1, r)$ is $r + 1$, hence there are $(r + 1)/3$ orbits).

Let also ω be a primitive element of F_r, that is an element such that $\omega^j \neq 1$, for any $j = 1, 2, \ldots, r-2$. Then, Beth's result[6;7] states that the partitions induced by multiplying and translating O by specific values λ, g as $\lambda \cdot O + g$ form a 1-factorization of F_r. Here $\lambda \cdot O + g$ means that we take each of the hyperedges $A_i \in O$, and transform their elements affinely into $\lambda \cdot A_i + g$, thus obtaining another hyperedge. Specifically, λ needs to take the values of the powers of ω given by $\lambda = \omega^j$, $j = 1, \ldots, (r-1)/2$, and g can take any value in F_r.

The key for us is that this construction can be evaluated very efficiently, by simply iterating over the orbits of π and the values λ, g.

Example: $r = 5$. For $m = 6$ pools, $r = m - 1 = 5$ is a prime number and Beth's construction applies. We go through the construction in this setting as an illustrative example.

1. For $r = 5$, we have $F_r = \{0, 1, 2, 3, 4\}$ and $\text{PG}(1, r) = \{0, 1, 2, 3, 4, \infty\}$.

2. We compute orbits of π by repeatedly applying π as follows:

$$\pi(0) = -\left(\frac{1+0}{0}\right) \equiv \infty, \qquad \pi(\infty) = -\left(\frac{1+\infty}{\infty}\right) \equiv 4, \qquad \pi(4) = -\left(\frac{1+4}{4}\right) \equiv 0,$$
$$\pi(1) = -\left(\frac{1+1}{1}\right) \equiv 3, \qquad \pi(3) = -\left(\frac{1+3}{3}\right) \equiv 2, \qquad \pi(2) = -\left(\frac{1+2}{2}\right) \equiv 1.$$

So we have the two orbits $O = \{\{0, \infty, 4\}, \{1, 3, 2\}\}$ that partition $\mathrm{PG}(1, r)$.

3. We find a primitive element of F_r by looking at powers

$$1^1 = 1, \qquad 2^1 = 2, \qquad 3^1 = 3, \qquad 4^1 = 4,$$
$$1^2 = 1, \qquad 2^2 = 4, \qquad 3^2 = 4, \qquad 4^2 = 1,$$
$$1^3 = 1, \qquad 2^3 = 3, \qquad 3^3 = 2, \qquad 4^3 = 4,$$
$$1^4 = 1, \qquad 2^4 = 1, \qquad 3^4 = 1, \qquad 4^4 = 1.$$

So we can choose $\omega \in \{2, 3\}$. We will (arbitrarily) choose $\omega = 2$.

4. Finally, we loop through λ and g. For $\omega = 2$, λ loops through $\{2, 4\}$.

	$\lambda = 2$	$\lambda = 4$
$g = 0$	$\{0, \infty, 3\}$ $\{2, 1, 4\}$	$\{0, \infty, 1\}$ $\{4, 2, 3\}$
$g = 1$	$\{1, \infty, 4\}$ $\{3, 2, 0\}$	$\{1, \infty, 2\}$ $\{0, 3, 4\}$
$g = 2$	$\{2, \infty, 0\}$ $\{4, 3, 1\}$	$\{2, \infty, 3\}$ $\{1, 4, 0\}$
$g = 3$	$\{3, \infty, 1\}$ $\{0, 4, 2\}$	$\{3, \infty, 4\}$ $\{2, 0, 1\}$
$g = 4$	$\{4, \infty, 2\}$ $\{1, 0, 3\}$	$\{4, \infty, 0\}$ $\{3, 1, 2\}$

Note that each cell in the resulting output table forms a partition of $\{0, 1, 2, 3, 4, \infty\}$ as desired. Looping over the cells and using the partitions to form pools yields the desired design.

S5 Relationship to design theory

Here we describe how the HYPER design fits in the broader context of design theory. See Beth et al.[8;9] for an excellent introduction to relevant design theory; we will follow the notation and terminology from those references.

For us, a design I is a collection of points V and blocks B, and an assignment of some points to some blocks. In group testing, the points correspond to individuals (or more generally, to samples), and the blocks correspond to pools. The terminology of points and blocks is meant to be evocative of geometry, and indeed designs are closely connected to finite geometries such as affine and projective planes. Intuitively, points can sometimes be viewed as geometric points, while blocks can be viewed as lines. Points will be denoted with lowercase letters such as p, while blocks will be denoted with upper case letters such as B. The fact that point p is associated with (or incident on) block B is denoted as pIB.

Designs are called q-hypergraphs, if the size of the set of points incident on each block B is q, i.e., $(B) = \{p \in V : pIB\}$ has size q. For group testing, this means that each sample is assigned to q pools. We are thus interested in q-hypergraphs, for small values of q, such as $2, 3$.

A partition of a design is a disjoint union of its blocks into parts. A parallel class of a design is a collection of blocks such that each point is incident on exactly one block. This is analogous of the geometric idea that parallel lines do not intersect, and so if we partition the space into parallel lines, then each point belongs to exactly one such line. A design is called resolvable if it has a partition into parallel classes (a.k.a 1-factorization, parallelism or resolution).

For group testing, a resolution means that in each part of the partition of the blocks, we use each pool exactly once. Since our goal is to use pools in a balanced way, this is precisely what we want. Thus, from a design theory perspective, we are interested exactly in resolutions of q-hypergraphs. There is a great amount of work on existence and constructions of such resolutions, see, e.g., Ch VIII of Beth et al.[8] and references therein.

One classical strategy is the permutation group action approach. Here, we start with a collection $\{B_j\}$, $j = 1, \dots, J$ of base blocks (say of size q), viewed as subsets of the set of pools $[m] = \{1, \dots, m\}$ where m is the number of pools, and a subgroup G of the permutation group S_m on m elements. If the action of G on $B_j \subset [m]$ $j = 1, \dots, J$ leads to J non-overlapping orbits, then there are classical conditions under which the collection of these orbits forms a t-design (i.e., a hypergraph such that all t-subsets of points are incident on the same number of blocks), see Theorem III.8.2 on p. 207 of Beth et al.[8]. A more specific technique is the difference cycle method, which is an application of the permutation group action method when the cyclic group $G = \mathbb{Z}_l$ acts on the vertices $V = \mathbb{Z}_l$ by translation. This approach leads to resolutions of the complete hypergraph for both $q = 2$ and $q = 3$, see Section VIII.8 of Beth et al.[8]. These are precisely the algorithms that we use.

S6 Reordering HYPER pool designs to aid lab implementation

To aid lab implementation, our online tool (`http://hyper.covid19-analysis.org`) presents designs after carrying out a re-ordering that we describe here. As we illustrated in Fig. 1, HYPER assigns individuals to pools by cycling through a sequence of pool assignments given by hypergraph factorization. Namely, for $q = 2$ splits and $m = 6$ pools labelled A-F, we cycled through the 15 possible pairs of pools in the order: AB, CD, EF, BC, DF, AE, BD, AF, CE, BE, CF, AD, BF, DE, AC. Namely, individual 1 was assigned to pools A and B, individual 2 to C and D, and so on; after individual 15, we return to the beginning of the sequence and cycle through again. For $n = 18$ individuals, this would result in the following pool assignments:

Individual	1	2	3	4	5	6	7	8	9	10	11	12	13	14	15	16	17	18
Pools	AB	CD	EF	BC	DF	AE	BD	AF	CE	BE	CF	AD	BF	DE	AC	AB	CD	EF

For use in a lab protocol, it can sometimes be helpful to re-order these assignments so that individuals assigned to the same pair of pools appear consecutively:

Individual	1	2	3	4	5	6	7	8	9	10	11	12	13	14	15	16	17	18
Pools	AB	AB	CD	CD	EF	EF	BC	DF	AE	BD	AF	CE	BE	CF	AD	BF	DE	AC

This is useful if we plan to first combine the samples from individuals 1 and 2, then split that combined sample into pools A and B. Likewise for individuals 3 and 4, as well as 5 and 6, in this case. To avoid confusion, we emphasize that we have simply re-ordered the pool assignments to show the repeated ones one after the other. Thus, the table does not show AB, CD and EF as the first three pairs, but rather AB is repeated twice, then CD is repeated twice, and so on.

S7 Correcting for false negatives in HYPER

The conservative decoder used in HYPER does not correct for any pooled tests with false negative results, in contrast to, e.g., P-BEST[3]. Such error correction may be incorporated by introducing a tolerance so that individuals in just one or two negative pools are still considered putative positives, as has been previously done for random assignment[1]. Doing so can improve the overall sensitivity of HYPER. However, note that an important source of false negatives is the dilution of viral loads below the limit of detection. An individual with small viral load is hence likely to yield false negatives in most of its pools, in which case error-correction may not significantly improve sensitivity. It is likely most useful for correcting false negative PCR results that are independent across tests, e.g., as may arise while performing the testing procedure. Note also that it can dramatically reduce efficiency. In our illustrative example (Fig. 1), allowing a tolerance of one negative pool would turn individuals 1, 5, and 9-12 into putative positives. Labs must consider how to properly weigh these tradeoffs according to their specific needs and their anticipated sources of errors.

S8 Balanced array designs

In addition to plate-based arrays[4], we consider balanced array designs. In particular, the plate-based arrays[4] proposed for COVID-19 testing use 8×12 and 16×24 arrays. This corresponds to plate sizes common in laboratory environments, making these choices convenient in practice. However, it results in imbalanced pools (Table 1) because the row pools are larger than the column pools. To address this, one could instead use square arrays, though these may no longer corresponds to common physical plate sizes in the lab. In these cases, the arrays become primarily a conceptual tool for constructing the design. In particular, taking $k = m/2$, one could use a $k \times k$ array with $n = k^2 = (m/2)^2$ individuals. This design has balanced pools since all pools contain k individuals. However, this is somewhat inflexible since n must then be a perfect square. One way to address this is to partially fill a larger array design than needed, i.e., consider a square array with holes.

For example, to replace the 8×12 array, which covers $n = 96$ individuals, one could use a 10×10 array with four holes. Note, however, that the holes must be placed carefully to preserve maximal balance of the pools. For example, if the holes are all placed on the first row, then that row pool will have four fewer individuals than the rest of the row pools. Instead, the holes should be placed along diagonals of the array. In this way, the pools in the array stay maximally balanced.

Taking this approach for general n corresponds to filling the array along the diagonals. For $k = 3$, e.g., individuals may be incrementally filled into a 3×3 array as follows:

	D	E	F
A	1		
B		2	
C			3

$\longrightarrow$

	D	E	F
A	1	4	
B		2	5
C	6		3

$\longrightarrow$

	D	E	F
A	1	4	7
B	8	2	5
C	6	9	3

Namely, we assign individuals to cells (and the corresponding pools) as follows:

Individual	1	2	3	4	5	6	7	8	9
Cell	(0,0)	(1,1)	(2,2)	(0,1)	(1,2)	(2,0)	(0,2)	(1,0)	(2,1)
Pools	AD	BE	CF	AE	BF	CD	AF	BD	CE

i.e., the sequence of assignments to cells are generated for general k as

$$(i, i + \Delta_1 \bmod k) \text{ for } i = 0, \dots, k-1;\ \Delta_1 = 0, \dots, k-1,$$

and the pool assignments are given by the rows and columns.

This approach is limited to producing designs with at most k^2 individuals. However, it can be extended to allow for more individuals, i.e., to create designs with $n > k^2$, by going back to the start and placing multiple individuals in each cell. Namely, individual $k^2 + 1$ is placed in the same cell as individual 1, individual $k^2 + 2$ is with individual 2, and so on. This parallels how HYPER handles $n > \binom{m}{q}$ individuals. Here it produces a design with maximally balanced pools. Pool combinations are not maximally balanced since some combinations are unused while others are assigned twice, but they are maximally balanced among those that are used.

The design can also be extended to three-dimensional arrays that assign each individual to $q = 3$ pools. For $k = 3$, e.g., we have the following $3 \times 3 \times 3$ array with pools along each slice (pool B shaded in to illustrate):

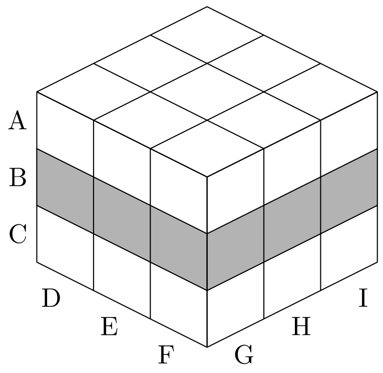

The sequence of assignments to cells are now generated as

$$(i, i + \Delta_1 \bmod k, i + \Delta_1 + \Delta_2 \bmod k) \text{ for } i = 0, \ldots, k-1;\ \Delta_1 = 0, \ldots, k-1;\ \Delta_2 = 0, \ldots, k-1,$$

yielding the following pool assignments for $k = 3$:

Individual	1	2	3	4	5	6	7	8	9
Cell	(0,0,0)	(1,1,1)	(2,2,2)	(0,1,1)	(1,2,2)	(2,0,0)	(0,2,2)	(1,0,0)	(2,1,1)
Pools	ADG	BEH	CFI	AEH	BFI	CDG	AFI	BDG	CEH

Individual	10	11	12	13	14	15	16	17	18
Cell	(0,0,1)	(1,1,2)	(2,2,0)	(0,1,2)	(1,2,0)	(2,0,1)	(0,2,0)	(1,0,1)	(2,1,2)
Pools	ADH	BEI	CFG	AEI	BFG	CDH	AFG	BDH	CEI

Individual	19	20	21	22	23	24	25	26	27
Cell	(0,0,2)	(1,1,0)	(2,2,1)	(0,1,0)	(1,2,1)	(2,0,2)	(0,2,1)	(1,0,2)	(2,1,0)
Pools	ADI	BEG	CFH	AEG	BFH	CDI	AFH	BDI	CEG

Analogous to the $q = 2$ balanced arrays, this approach is limited to producing designs with at most k^3 individuals but can be extended by cycling back through the pool assignments as is done in HYPER.

These extensions make it possible to generate array designs with parameters matching those considered for HYPER (Table 2), allowing for further comparison with HYPER.

S9 Reed-Solomon Kautz-Singleton code-based designs

We also consider the celebrated Kautz-Singleton[13] construction that converts a b-nary code (often a Reed-Solomon[14] code) into a binary design matrix. This design has recently been shown to be order optimal for the so-called probabilistic group testing problem under certain assumptions[15].

For the reader's convenience, here we detail the steps involved in constructing the Reed-Solomon Kautz-Singleton (RS-KS) design used in this paper in our notation. The design is parameterized by (n, m, q), together with an additional parameter f used in the construction. The first step is to construct a m/q-nary matrix from a Reed-Solomon code as follows:

1. Let $b \coloneqq m/q$, and let F_b be the finite field of size b (so b must be a prime power).

2. Let x be a generator of F_b, i.e., $F_b = \{0, 1, x, \ldots, x^{b-2}\}$, with $x^{b-1} = 1$.

3. Let $\mathbf{a}_1, \ldots, \mathbf{a}_{b^f} \in F_b^f$ be an enumeration of F_b^f, e.g., in lexicographic order:

$$\begin{array}{llll}
\mathbf{a}_1 = (0,0,0,\ldots,0) & \mathbf{a}_{b+1} = (0,1,0,\ldots,0) & \cdots & \mathbf{a}_{b^{f-1}+1} = (0, x^{b-2}, x^{b-2}, \ldots, x^{b-2}) \\
\mathbf{a}_2 = (1,0,0,\ldots,0) & \mathbf{a}_{b+2} = (1,1,0,\ldots,0) & \cdots & \mathbf{a}_{b^{f-1}+2} = (1, x^{b-2}, x^{b-2}, \ldots, x^{b-2}) \\
\mathbf{a}_2 = (x,0,0,\ldots,0) & \mathbf{a}_{b+2} = (x,1,0,\ldots,0) & \cdots & \mathbf{a}_{b^{f-1}+2} = (x, x^{b-2}, x^{b-2}, \ldots, x^{b-2}) \\
\vdots & \vdots & \cdots & \vdots \\
\mathbf{a}_b = (x^{b-2},0,0,\ldots,0) & \mathbf{a}_{2b} = (x^{b-2},1,0,\ldots,0) & \cdots & \mathbf{a}_{b^f} = (x^{b-2}, x^{b-2}, x^{b-2}, \ldots, x^{b-2})
\end{array}$$

4. Form the $q \times b^f$ matrix with b-nary entries:

$$\begin{bmatrix} P_{\mathbf{a}_1}(0) & \cdots & P_{\mathbf{a}_{b^f}}(0) \\ P_{\mathbf{a}_1}(1) & \cdots & P_{\mathbf{a}_{b^f}}(1) \\ P_{\mathbf{a}_1}(x) & \cdots & P_{\mathbf{a}_{b^f}}(x) \\ \vdots & & \vdots \\ P_{\mathbf{a}_1}(x^{q-2}) & \cdots & P_{\mathbf{a}_{b^f}}(x^{q-2}) \end{bmatrix} \in F_b^{q \times b^f},$$

   where $P_{\mathbf{a}}(z) \coloneqq \sum_{i=0}^{f-1} a_i z^i$ for any $\mathbf{a} = (a_0, \ldots, a_{f-1}) \in F_b^f$ and any $z \in F_b$.

   This requires that $q \leq b$ (so the evaluation is at distinct elements), and $f \leq q$ (so it is injective; we need this to be a meaningful code).

The second step, the Kautz-Singleton construction, is to convert the $b = m/q$-nary matrix into a binary design matrix by

replacing each letter of the code by a binary column vector $\{0,1\}^b$ with a one-hot encoding, i.e.,

$$\begin{aligned}
0 &\mapsto (1,0,0,\dots,0,0) \in \{0,1\}^b,\\
1 &\mapsto (0,1,0,\dots,0,0) \in \{0,1\}^b,\\
x &\mapsto (0,0,1,\dots,0,0) \in \{0,1\}^b,\\
&\vdots\\
x^{b-2} &\mapsto (0,0,0,\dots,0,1) \in \{0,1\}^b.
\end{aligned}$$

This gives a $qb \times b^f$ binary design matrix with q ones in each column. Since $m = qb$, the size of the matrix is equivalently $m \times (m/q)^f$.

Note that this approach is limited to producing designs for which

- $f \leq q$.
- $n = (m/q)^f$
- $m/q \geq q$, i.e., $m \geq q^2$, and
- m/q is a prime power.

Note that the requirement that $f \leq q$, means that for the choices $q = 2$ and $q = 3$ we consider, the relevant designs are: $(q = 2, f = 2)$, $(q = 3, f = 2)$, and $(q = 3, f = 3)$.

For $n \leq (m/q)^f$, we consider truncating the design by taking the first n columns to obtain the desired $m \times n$ binary design matrix. The m rows correspond to the m pools, the n columns correspond to the n individuals, and the q ones in each column identify to which pools each individual is assigned.

For $n > (m/q)^f$, we consider extending the design in a straightforward way by recycling the pool assignments, i.e., by concatenating the design matrix with copies on the right until it has at least n columns total. Namely, individual $(m/q)^f + 1$ is assigned the same pools as individual 1, individual $(m/q)^f + 2$ is assigned the same pools as individual 2, and so on. This parallels how HYPER handles $n > \binom{m}{q}$ individuals.

The resulting design parameters from Table 2 that can be handled by this construction are indicated there by asterisks ($*$). We found that for all these designs, the pools were maximally balanced and the pool combinations were maximally balanced among those that were used. When m/q is not a prime power, we considered using a prime power b larger than m/q in the construction then dropping $qb - m$ rows to obtain m final rows, but doing so produced columns with less than q ones (i.e., those individuals were not placed in q pools). This could be addressed by removing those columns, but in the cases we tried, this led to the pools being unbalanced.

Example. We work out the construction for $n = 9, m = 6, q = 2, f = 2$ as an illustrative example. Here, $b = 3$ so we have $F_3 = \{0, 1, 2\}$, i.e., $(\mathbb{Z}_3, +, \cdot)$, with generator $x = 2$. Next, we have the enumeration

$$\mathbf{a}_1 = (0,0) \qquad \mathbf{a}_4 = (0,1) \qquad \mathbf{a}_7 = (0,2)$$
$$\mathbf{a}_2 = (1,0) \qquad \mathbf{a}_5 = (1,1) \qquad \mathbf{a}_8 = (1,2)$$
$$\mathbf{a}_3 = (2,0) \qquad \mathbf{a}_6 = (2,1) \qquad \mathbf{a}_9 = (2,2)$$

which yields the following b-nary matrix

$$\begin{bmatrix} P_{\mathbf{a}_1}(0) & \cdots & P_{\mathbf{a}_9}(0) \\ P_{\mathbf{a}_1}(1) & \cdots & P_{\mathbf{a}_9}(1) \end{bmatrix} = \begin{bmatrix} 0 & 1 & 2 & 0 & 1 & 2 & 0 & 1 & 2 \\ 0 & 1 & 2 & 1 & 2 & 0 & 2 & 0 & 1 \end{bmatrix} \in F_3^{2\times 9}.$$

Converting to a binary matrix finally yields the following design:

	1	2	3	4	5	6	7	8	9
A	1	0	0	1	0	0	1	0	0
B	0	1	0	0	1	0	0	1	0
C	0	0	1	0	0	1	0	0	1
D	1	0	0	0	0	1	0	1	0
E	0	1	0	1	0	0	0	0	1
F	0	0	1	0	1	0	1	0	0

Note that this has balanced pools and maximally balanced pool combinations, but does not use all pool pairs (e.g., the pool pair AB is unused).

S10 Performance characterization under a common statistical model

Here we study the performance of HYPER under the common statistical model where each individual (or in more general contexts, each sample) is positive independently at random with probability p and each test may be incorrect with some probability, i.e., each test has a specificity of $1-\alpha$ and a sensitivity of β. We will use T to denote the number of tests used by HYPER (including stage 2 tests). Note that this is a random variable due to randomness in both which individuals are positive and in any test errors.

S10.1 An upper bound on the expected number of tests in the noiseless case

We first consider the noiseless case, and give an upper bound on the expected number of tests used by HYPER (including stage 2 tests). To get this result, we leverage the Dawson-Sankoff inequality[16], a nontrivial refinement of the Bonferroni union-intersection inequalities, which we use in the form given by Galambos[17]. We will later relax the assumption made here that tests have perfect sensitivity and specificity.

Theorem 1 (Performance in the noiseless case): Suppose that all tests are noiseless ($\alpha = 0$, $\beta = 1$), and consider a HYPER design with n individuals, m pools, and q splits, such that n is a multiple of m/q. For any integer $l \geq 2$, the expected number of tests is upper bounded as follows:

$$\mathbb{E}(T) \leq m + n \cdot \left[1 - \frac{2q}{l}(1-p)^k + \frac{q(q-1)}{l(l-1)}(1-p)^{2k-u}\right]. \tag{1}$$

where $k = nq/m$ is the pool size and $u = \binom{m-2}{q-2} \cdot \lceil n/\binom{m}{q} \rceil$. The bound becomes an equality when (A) $q = 1$, (B) $n < \binom{m}{q}$ and $q = 2$; and with taking $l = 2$ in both cases, or when (C) n is a multiple of $\binom{m}{q}$. For general q, the optimal choice for the parameter l is bounded by $l \leq \lfloor (q-1)(1-p)^{k-u} \rfloor + 2$.

Note that in cases (A) and (B), the bound (1) not only becomes an equality but can also be simplified by using the fact that $u = 0$ in case (A) and $u = 1$ in case (B), yielding the following expressions:

$$\text{Case (A)}: \; \mathbb{E}(T) = m + n \cdot \left[1 - (1-p)^k\right], \qquad \text{Case (B)}: \; \mathbb{E}(T) = m + n \cdot \left[1 - 2(1-p)^k + (1-p)^{2k-1}\right],$$

which can be combined into a single formula as follows:

$$\text{Case (A) or (B)}: \quad \mathbb{E}(T) = m + n \cdot \left[p + r\left(1 - r^{k-1}\right)^q\right],$$

where $r = 1 - p$.

Proof of Theorem 1. Let R_i, $i = 1, \ldots, n$ be the indicator of the event that we need to retest individual i in stage 2. Using the standard approach of calculating $\mathbb{E}(T)$, we find that the number of tests required is equal to m (one for each pool), plus any retests required. Hence,

$$\mathbb{E}(T) = m + \sum_{i=1}^{n} \Pr(R_i).$$

Now, for our decoder, R_i happens precisely when all groups containing i are positive. Let X_i, $i = 1, \ldots, n$ be the indicator that the i-th individual is positive. Let $G_j \subset \{1, \ldots, n\}$ be the individuals contained in pool j, for $j = 1, \ldots, m$.

We use a refined version of the Bonferroni inequality known as the Dawson-Sankoff inequality [16] to bound $\Pr(R_i)$. First let us recall the familiar Bonferroni union-intersection inequalities. Consider events $A_1, \ldots, A_N$, and for all $j \in \{1, \ldots, N\}$, define

$$S_j = \sum_{i_1 < i_2 < \ldots < i_j} \Pr(A_{i_1} \cap \cdots \cap A_{i_j}).$$

Then, the well-known Bonferroni inequalities state that for even $h \in \{1, \ldots, N\}$,

$$\Pr(\cup_{j=1}^{N} A_j) \geq \sum_{j=1}^{h} (-1)^{j-1} S_j.$$

By construction, there are q pools containing individual i. Without loss of generality, we can assume by relabeling the pools that their indices are $1, \ldots, q$. Then, the individuals contained in them are $G_1, \ldots, G_q$; which implicitly depend on i, but this is not displayed for notational simplicity. In the Bonferroni inequality, we set $N = q$, and for $j = 1, \ldots, q$,

$$A_j = \{\forall\, k \in G_j, X_k = 0\}$$

be the event that all individuals contained in the j-th pool containing individual i test negative. As above, these implicitly depend on i, but this is not displayed for notational simplicity. By definition, the i-th individual is not retested, so R_i does not happen, precisely when none of the individuals contained in the pools $1, \ldots, q$ to which individual i belongs to are positive. Equivalently, $\neg R_i = \cup_{j=1}^{q} A_j$. Then for any even integer h

$$\Pr(\neg R_i) = \Pr(\cup_{j=1}^{q} A_j) \geq \sum_{j=1}^{h} (-1)^{j-1} S_j.$$

Taking $h = 2$, we thus find

$$\Pr(R_i) \leq 1 - S_1 + S_2.$$

We can get sharper results with the Dawson-Sankoff inequality [16]. In the form given by Galambos [17], this states that for any integer $l \geq 2$,

$$\Pr(\cup_{j=1}^{q} A_j) \geq \frac{2S_1}{l} - \frac{2S_2}{l(l-1)}.$$

Now we can write $\Pr(A_j) = (1-p)^{|G_j|}$ and for $j \neq j'$, we have $\Pr(A_j \cap A_{j'}) = (1-p)^{|G_j \cup G_{j'}|}$.

It remains to bound $|G_j|$. Here HYPER designs are useful, because they try to balance $|G_j|$. In each consecutive block of m/q individuals, they use each of the m pools exactly once. Thus, in each G_j, there is at most one new individual. Recall that k is the number of consecutive blocks of individuals of size m/q, and we assumed that $k = nq/m$ is an integer. Based on the above, we have $|G_j| = k$.

Moreover, $|G_j \cup G_{j'}| = |G_j| + |G_{j'}| - |G_j \cap G_{j'}|$, and the intersection $G_j \cap G_{j'}$ has size at most $|G_j \cap G_{j'}| \leq u := \binom{m-2}{q-2} \cdot \lceil n / \binom{m}{q} \rceil$. The reason is that, since HYPER designs are maximally balanced, they only intersect at most $\binom{m-2}{q-2}$

times in every consecutive block of $\binom{m}{q}$ individuals. These intersections correspond to the number of ways of choosing the remaining $q-2$ pools out of the remaining $m-2$. Hence, $\Pr(A_j) = (1-p)^k$ and for $j \neq j'$, $\Pr(A_j \cap A_{j'}) \leq (1-p)^{2k-u}$. Thus, $S_1 \geq q(1-p)^k$ and $S_2 \leq \binom{q}{2}(1-p)^{2k-u}$. In addition, we have equality for S_2 when either (A) $q = 1$, in which case the intersection is empty and $u = 0$, or (B) $n < \binom{m}{q}$ and $q = 2$ (in which case we know that the groups intersect in exactly $u = 1$ individual, which is the individual defining them) or (C) n is a multiple of $\binom{m}{q}$ (in which case we know that the number of intersections is exactly given by u for each pair of groups). This leads to the desired result.

As is known[16;17], the optimal choice for l is $l = \lfloor 2S_2/S_1 \rfloor + 2$. We can approximate the optimal choice using the calculations from the proof as

$$l \leq \left\lfloor \frac{2\binom{q}{2}(1-p)^{2k-u}}{q(1-p)^k} \right\rfloor + 2 \leq \left\lfloor (q-1)(1-p)^{k-u} \right\rfloor + 2.$$

This finishes the proof.

S10.2 Optimal efficiency for HYPER with $q = 2$

Our next result characterizes the optimal efficiency of HYPER designs with $q = 2$ for noiseless tests in the case where the number of individuals per batch is large and the prevalence is small.

Proposition 2 (Optimal efficiency for HYPER with $q = 2$): Suppose that all tests are noiseless ($\alpha = 0$, $\beta = 1$), and consider the expected number of tests per individual $\mathbb{E}(T)/n$ for $q = 2$ HYPER designs in the limit of large batches $n \to \infty$, where the number of pools per individual $y = m/n$ is fixed. For this asymptotic performance, the optimal number of pools per individual is approximately $y \approx 2p^{2/3} - p$ in the limit of diminishing prevalence ($p \to 0$) with a corresponding expected tests per individual of approximately $\mathbb{E}(T)/n \approx 3p^{2/3}$.

In comparison, the optimal expected tests per individual for Dorfman testing is approximately $2p^{1/2}$ for small p (see, e.g., Finucan[18]). HYPER designs with $q = 2$ improve on Dorfman designs in this regime. The expected tests per individual for three-stage-testing[18] and double-pooling[2] are approximately $3p^{2/3}$ in the limit. This matches HYPER, but note that HYPER is a two-stage deterministic approach. The expected tests per individual for the hierarchical testing methods proposed in Finucan[18] and Mutesa et al.[19] is approximately $ep\ln(1/p)$ as $p \to 0$, which is asymptotically more efficient, but note that these methods use multiple stages.

More generally, there is a lot of work on optimality for group testing, for various cases, e.g., two-stage and multi-stage algorithms, adaptive and non-adaptive algorithms, in worst case or average case, etc[20–25]. As $p \to 0$, these works and others discuss a universal lower bound of order $\Theta(p\ln(1/p))$ on the expected tests per individual. However, the best efficiency (and the algorithms that achieve it) depends on the specific rate at which $p \to 0$. In particular, under the same statistical model as in our paper, Mezard and Toninelli[23] construct certain tests where each sample is placed into $q = \lceil \ln(1/p)/\ln 2 \rceil$ pools, and show that these attain an asymptotic expected tests per individual of $p\ln(1/p)$. Coja-Oghlan et al.[26] constructs a 2-stage algorithm with asymptotically optimal efficiency, requiring $q = m\ln(2)/(np)$. Gebhard et al.[27] discusses similar proposals for the noisy case. In our work, the constraints we work with do not allow q to grow with $p \to 0$. Scarlett[28] proposes a 4-stage algorithm with asymptotically optimal efficiency. Our work is also related to

d-disjunctive superimposed matrices for pooled testing, which work without errors when the number of positives is at most d and there are no false positives[13;29]. Sharp bounds on the size of d-disjunctive matrices were constructed in Erdös et al.[30]. Classic error-correcting codes such as Reed-Solomon codes were suggested for group testing dating back at least to Kautz and Singleton[13].

Proof of Proposition 2. Taking the limit as $n \to \infty$ with $m/n = y$ fixed and noting that $n < \binom{m}{2}$ eventually in this limit, we obtain that eventually

$$E = \frac{\mathbb{E}(T)}{n} = \frac{m}{n} + 1 - 2(1-p)^{2n/m} + (1-p)^{4n/m-1} = y + 1 - 2(1-p)^{2/y} + (1-p)^{4/y-1}.$$

To optimize, we differentiate E with respect to y, obtaining

$$\frac{\partial E}{\partial y} = 1 + \frac{4(1-p)^{2/y}\log(1-p)}{y^2} - \frac{4(1-p)^{4/y-1}\log(1-p)}{y^2}.$$

The optimal y can be obtained by solving $0 = \partial E/\partial y$ for y in terms of p. We approximate this solution in the limit $p \to 0$ by taking the leading two terms of the Puiseux expansion (around $p = 0$) of the degree four Taylor approximation of $\partial E/\partial y$ (with respect to $p = 0$). This has one branch corresponding to a real solution yielding $y^\star \approx 2p^{2/3} - p$.

Substituting $y = 2p^{2/3} - p$ into E and computing a Taylor approximation yields

$$E^\star = 2p^{2/3} - p + 1 - 2(1-p)^{2/(2p^{2/3}-p)} + (1-p)^{4/(2p^{2/3}-p)-1} = 3p^{2/3} + O(p^{4/3}) \approx 3p^{2/3},$$

completing the derivation.

S10.3 Generalization to the noisy case

We next study the noisy case, where each of the pooled tests can have false positives and false negatives. Our first result gives an exact formula for the expected number of tests for $q \leq 2$, and moreover, also gives formulas for the sensitivity, specificity, true negative probability, and true positive probability. Our second result gives a more generally applicable upper bound on the expected number of tests that is valid for all q.

Theorem 3 (Performance in the noisy case for $q \leq 2$): Suppose each test has specificity $1-\alpha$ and sensitivity β, and consider a HYPER design with n individuals, m pools, and q splits, such that n is a multiple of m/q. Suppose further that $q \leq 2$ and $n \leq \binom{m}{q}$. Then the expected number of tests has the following exact form:

$$\mathbb{E}(T) = m + n\,(p \cdot \beta^q + r \cdot \gamma)\,, \tag{2}$$

where $k = nq/m$, $r = 1-p$, and $\gamma = [\beta + (\alpha - \beta)\cdot r^{k-1}]^q$. The overall sensitivity and specificity are, respectively,

$$\Pr(\widehat{X}_i = 1 | X_i = 1) = \beta^{q+1}, \qquad \Pr(\widehat{X}_i = 0 | X_i = 0) = 1 - \alpha\gamma, \tag{3}$$

where X_i denotes the true status of individual i and $\widehat{X}_i$ is the status declared by HYPER. Finally, the true negative and true positive probabilities are, respectively,

$$\Pr(X_i = 0 | \widehat{X}_i = 0) = \frac{1}{1 + o^{-1}\frac{1-\beta^{q+1}}{1-\alpha\gamma}}, \qquad \Pr(X_i = 1 | \widehat{X}_i = 1) = \frac{1}{1 + o\frac{\alpha\gamma}{\beta^{q+1}}}, \tag{4}$$

where $o = (1-p)/p$ is the odds ratio.

Theorem 4 (Performance in the noisy case for all q): Suppose each test has specificity $1-\alpha$ and sensitivity β, and consider a HYPER design with n individuals, m pools, and q splits, such that n is a multiple of m/q. For any integer $l \geq 2$, the expected number of tests is upper bounded as follows:

$$\mathbb{E}(T) \leq m + n \cdot \left[1 - \frac{2q}{l}p_1 + \frac{q(q-1)}{l(l-1)}p_2\right], \tag{5}$$

where $p_1 = 1-\beta+(\beta-\alpha)r^k$, $p_2 = p_1^2 + (\beta-\alpha)^2 r^{2k-u}(1-r^u)$, $k = nq/m$, $r = 1-p$, and $u = \binom{m-2}{q-2} \cdot \lceil n/\binom{m}{q} \rceil$. The optimal choice of l, minimizing this upper bound, is achieved by $l = \lfloor (q-1)p_2/p_1 \rfloor + 2$.

Proof of Theorems 3 and 4. We will follow, to some extent, the notation and assumptions from Bilder's works (see, e.g., Bilder[31]). Let $\hat{H}_j$ be the binary result of testing group j, and H_j be the true status of the j-th group. Recall that $1-\alpha$ is the specificity of each grouped test, so $1-\alpha = \Pr(\hat{H}_j = 0|H_j = 0)$ (we use this notation in convention with the notion of α for the level of a test in hypothesis testing); likewise, β is the sensitivity (or power) of each grouped test, so $\beta = \Pr(\hat{H}_j = 1|H_j = 1)$. Moreover, each test outcome is independent. We have

$$\Pr(R_i) = \Pr(R_i|X_i = 1)\Pr(X_i = 1) + \Pr(R_i|X_i = 0)\Pr(X_i = 0) = \Pr(R_i|X_i = 1)\cdot p + \Pr(R_i|X_i = 0)\cdot(1-p).$$

The key is to determine the probabilities $\Pr(R_i|X_i = t)$. Now R_i happens if and only if each of the groups containing i have a positive status, i.e., for all $i \in G_j$, we have $\hat{H}_j = 1$:

$$R_i = \bigcap_{i\in G_j} \{\hat{H}_j = 1\}.$$

Since $q = 1, 2$, and $n \leq \binom{m}{q}$, these groups are non-overlapping outside of i, and thus, their probabilities are independent conditional on i. We can thus write

$$\Pr(R_i|X_i = t) = \Pr(\cap_{i\in G_j}\{\hat{H}_j = 1\}|X_i = t) = \prod_{i\in G_j} \Pr(\hat{H}_j = 1|X_i = t).$$

Next, we can condition on H_j for each term, to write

$$\begin{aligned}\Pr(\hat{H}_j = 1|X_i = t) &= \Pr(\hat{H}_j = 1|H_j = 1)\Pr(H_j = 1|X_i = t) + \Pr(\hat{H}_j = 1|H_j = 0)\Pr(H_j = 0|X_i = t)\\ &= \beta\cdot\Pr(H_j = 1|X_i = t) + \alpha\cdot\Pr(H_j = 0|X_i = t) = \beta + (\alpha-\beta)\cdot\Pr(H_j = 0|X_i = t).\end{aligned}$$

Moreover, since $H_j = \max_{i\in G_j} X_i$, we have

$$\Pr(H_j = 0|X_i = 1) = 0, \qquad \Pr(H_j = 0|X_i = 0) = \prod_{k\in G_j, k\neq i} \Pr(X_k = 0) = (1-p)^{|G_j|-1}.$$

Working our way back up, we can substitute these above to find

$$\Pr(\hat{H}_j = 1|X_i = 1) = \beta, \qquad \Pr(\hat{H}_j = 1|X_i = 0) = \beta + (\alpha-\beta)\cdot(1-p)^{|G_j|-1}.$$

Letting $n_i = |\{i : i \in G_j\}|$ be the number of groups that i belongs to, we find

$$\Pr(R_i|X_i = 1) = \beta^{n_i}, \qquad \Pr(R_i|X_i = 0) = \prod_{i\in G_j}[\beta + (\alpha-\beta)\cdot(1-p)^{|G_j|-1}].$$

Finally,

$$\Pr(R_i) = \beta^{n_i} \cdot p + \prod_{i \in G_j} [\beta + (\alpha - \beta) \cdot (1-p)^{|G_j|-1}] \cdot (1-p).$$

Hence

$$\begin{aligned}
\mathbb{E}(T) &= m + \sum_{i=1}^{n} \Pr(R_i) \\
&= m + \sum_{i=1}^{n} \left\{ \beta^{n_i} \cdot p + \prod_{i \in G_j} [\beta + (\alpha - \beta) \cdot (1-p)^{|G_j|-1}] \cdot (1-p) \right\} \\
&= m + p \cdot \sum_{i=1}^{n} \beta^{n_i} + (1-p) \cdot \sum_{i=1}^{n} \left\{ \prod_{i \in G_j} [\beta + (\alpha - \beta) \cdot (1-p)^{|G_j|-1}] \right\}.
\end{aligned}$$

Recall that for the hypergraph designs, $|G_j| \leq \lceil n/(m/q) \rceil$, and if n is an integer multiple of m/q as assumed here, then $|G_j| = nq/m$. Moreover, by construction, $n_i = q$. Hence we find

$$\mathbb{E}(T) = m + n \left\{ p \cdot \beta^q + (1-p) \cdot [\beta + (\alpha - \beta) \cdot (1-p)^{nq/m-1}]^q \right\}.$$

This gives the desired formula for the expected number of tests.

Next we derive the overall sensitivity, specificity, true negative probability, and true positive probability. Recall that $\widehat{X}_i$ is the indicator that the i-th individual is declared positive. This happens if all groups containing i are positive in the first round, and then the result of a second independent test $\widehat{X}_{i2}$ is also positive. Recall that X_i is the indicator that the i-th individual is positive, and $\Pr(X_i = 1) = p$. We are interested in the probabilities $\Pr(\widehat{X}_i = t | X_i = t)$ for $t \in \{0, 1\}$. Since the result of the retest is independent of the original tests and the retest has the same operating characteristics as any grouped test, we can write

$$\Pr(\widehat{X}_i = 1 | X_i = t) = \Pr(R_i = 1, \widehat{X}_{i2} = 1 | X_i = t) = \Pr(R_i = 1 | X_i = t) \cdot \Pr(\widehat{X}_{i2} = 1 | X_i = t)$$

We have $\Pr(\widehat{X}_{i2} = 1 | X_i = 0) = \alpha$ and $\Pr(\widehat{X}_{i2} = 1 | X_i = 1) = \beta$. Hence, using our previous results and denoting $\gamma = \prod_{i \in G_j} [\beta + (\alpha - \beta) \cdot (1-p)^{|G_j|-1}]$, we have $\Pr(\widehat{X}_i = 1 | X_i = 0) = \gamma \cdot \alpha$ and $\Pr(\widehat{X}_i = 1 | X_i = 1) = \beta^{n_i} \cdot \beta = \beta^{n_i+1}$. We are next interested in the probabilities $\Pr(X_i = t | \widehat{X}_i = \hat{t})$, for $t, \hat{t} \in \{0, 1\}$. For $\Pr(X_i = 1 | \widehat{X}_i = 1)$ this denotes the true positive probability; for $\Pr(X_i = 0 | \widehat{X}_i = 0)$ this denotes the true negative probability. Using Bayes' rule, we can write

$$\Pr(X_i = t | \widehat{X}_i = \hat{t}) = \frac{\Pr(\widehat{X}_i = \hat{t} | X_i = t) \Pr(X_i = t)}{\Pr(\widehat{X}_i = \hat{t})} = \frac{\Pr(\widehat{X}_i = \hat{t} | X_i = t) \Pr(X_i = t)}{(1-p) \cdot \Pr(\widehat{X}_i = \hat{t} | X_i = 0) + p \cdot \Pr(\widehat{X}_i = \hat{t} | X_i = 1)}.$$

Using our previous results yields

$$\begin{aligned}
\Pr(X_i = 1 | \widehat{X}_i = 0) &= \frac{\Pr(\widehat{X}_i = 0 | X_i = 1) \Pr(X_i = 1)}{(1-p) \cdot \Pr(\widehat{X}_i = 0 | X_i = 0) + p \cdot \Pr(\widehat{X}_i = 0 | X_i = 1)} = \frac{p \cdot [1 - \beta^{n_i+1}]}{(1-p) \cdot (1 - \alpha\gamma) + p \cdot [1 - \beta^{n_i+1}]}, \\
\Pr(X_i = 1 | \widehat{X}_i = 1) &= \frac{\Pr(\widehat{X}_i = 1 | X_i = 1) \Pr(X_i = 1)}{(1-p) \cdot \Pr(\widehat{X}_i = 1 | X_i = 0) + p \cdot \Pr(\widehat{X}_i = 1 | X_i = 1)} = \frac{p \cdot \beta^{n_i+1}}{(1-p) \cdot \alpha\gamma + p \cdot \beta^{n_i+1}}.
\end{aligned}$$

Recall that for the hypergraph designs, if n is an integer multiple of m/q, then $\gamma = [\beta + (\alpha - \beta) \cdot (1-p)^{nq/m-1}]^q$ and $n_i = q$. Hence, we find that the overall sensitivity and specificity are, respectively,

$$\Pr(\widehat{X}_i = 1 | X_i = 1) = \beta^{q+1}, \qquad \Pr(\widehat{X}_i = 0 | X_i = 0) = 1 - \alpha\gamma, \tag{6}$$

and, denoting the odds ratio as $o = (1-p)/p$, we find that the true negative and true positive probabilities are, respectively,

$$\Pr(X_i = 0|\widehat{X}_i = 0) = \frac{1}{1 + o^{-1}\frac{1-\beta^{q+1}}{1-\alpha\gamma}}, \qquad \Pr(X_i = 1|\widehat{X}_i = 1) = \frac{1}{1 + o\frac{\alpha\gamma}{\beta^{q+1}}}.$$

This finishes the proof of Theorem 3.

Now, we proceed to Theorem 4. This follows in a similar way to the previous Theorem 1, but with more involved calculations. As before, the Dawson-Sankoff inequality[16], in the form given by Galambos[17], states that for any integer l,

$$\Pr(\cup_{j=1}^{q} A_j) \geq \frac{2S_1}{l} - \frac{2S_2}{l(l-1)}.$$

Taking $A_j = \{\hat{H}_j = 0\}$, we find that the above equals $R_i^c = \cup_{j=1}^{q} A_j$. Now $S_1 = \sum \Pr(A_j)$, $S_2 = \sum_{j<g} \Pr(A_j \cap A_g)$. Thus it is enough to give a lower bound for $\Pr(\hat{H}_j = 0)$ and an upper bound for $\Pr(\hat{H}_j = \hat{H}_g = 0)$ for all $j \neq g$.

We can condition on H_j to write

$$\begin{aligned}
\Pr(\hat{H}_j = 0) &= \Pr(\hat{H}_j = 0|H_j = 0)\Pr(H_j = 0) + \Pr(\hat{H}_j = 0|H_j = 1)\Pr(H_j = 1) \\
&= (1-\alpha)\cdot\Pr(H_j = 0) + (1-\beta)\cdot[1 - \Pr(H_j = 0)] = 1 - \beta + (\beta - \alpha)\cdot\Pr(H_j = 0) \\
&= 1 - \beta + (\beta - \alpha)\cdot(1-p)^{|G_j|} = 1 - \beta + (\beta - \alpha)\cdot(1-p)^k.
\end{aligned}$$

In the last line, we have used that n is a multiple of m/q.

Similarly, we can calculate for $j \neq k$, noting that $|G_j| = |G_g| = k$, and denoting $|G_j \cap G_g| = v$, so that $|G_j \cup G_g| = 2k - v$,

$$\begin{aligned}
\Pr(\hat{H}_j = \hat{H}_g = 0) &= \sum_{a,b=0}^{1} \Pr(\hat{H}_j = \hat{H}_g = 0|H_j = a, H_g = b)\Pr(H_j = a, H_g = b) \\
&= (1-\alpha)^2\cdot r^{2k-v} + 2(1-\beta)(1-\alpha)r^k(1 - r^{k-v}) + (1-\beta)^2(1 - 2r^k + r^{2k-v}) \\
&= [1 - \beta + (\beta - \alpha)\cdot r^k]^2 + (\alpha - \beta)^2\cdot(r^{2k-u} - r^{2k}).
\end{aligned}$$

As discussed before, due to the construction of hypergraph factorization designs, we have $v \leq u = \binom{m-2}{q-2}\cdot\lceil n/\binom{m}{q}\rceil$. Since the above expression is monotonically increasing in u, we can also conclude that we can upper bound it by replacing v with u. This finishes the proof.

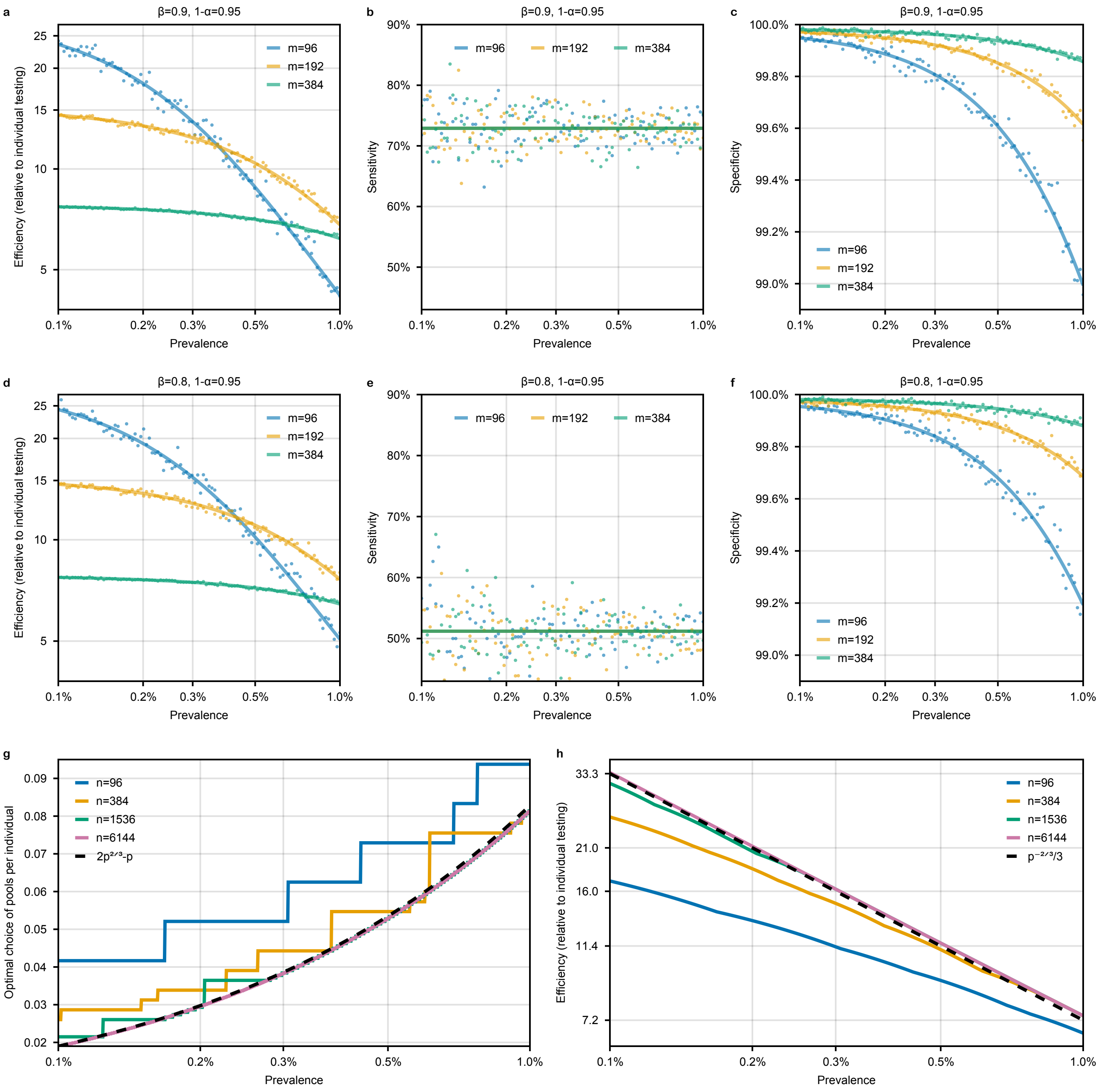

Supplementary Figure 1: Performance under a common statistical model. We consider the overall efficiency (**a**), sensitivity (**b**), and specificity (**c**) of three HYPER designs (three choices of m, all with $n = 3072$ and $q = 2$) under a general statistical model with noisy tests having sensitivity $\beta = 90\%$ and specificity $1 - \alpha = 95\%$. The theoretical predictions (solid curves) agree well with simulation results (scatter plots). For a lower sensitivity $\beta = 80\%$ (but same specificity $1 - \alpha = 95\%$), the efficiency (**d**) is slightly better (since more positives are missed), the sensitivity (**e**) is lower, and the specificity (**f**) is slightly improved (at larger prevalence). For noiseless tests ($\beta = 1 - \alpha = 1$), we study the optimal choice (**g**) of m for HYPER with $q = 2$ in the limit of large batches ($n \to \infty$) with diminishing prevalence ($p \to 0$). As n grows from $n = 96$ to $n = 6144$, the value of m optimizing the efficiency (found by exhaustive search) approaches the theoretical limit of $m/n \approx 2p^{2/3} - p$ for small prevalence p. Likewise, the corresponding efficiency (**h**; relative to individual testing) approaches $n/\mathbb{E}(T) \approx p^{-2/3}/3$ for small prevalence p. The approximation improves for increasingly small p as n grows.

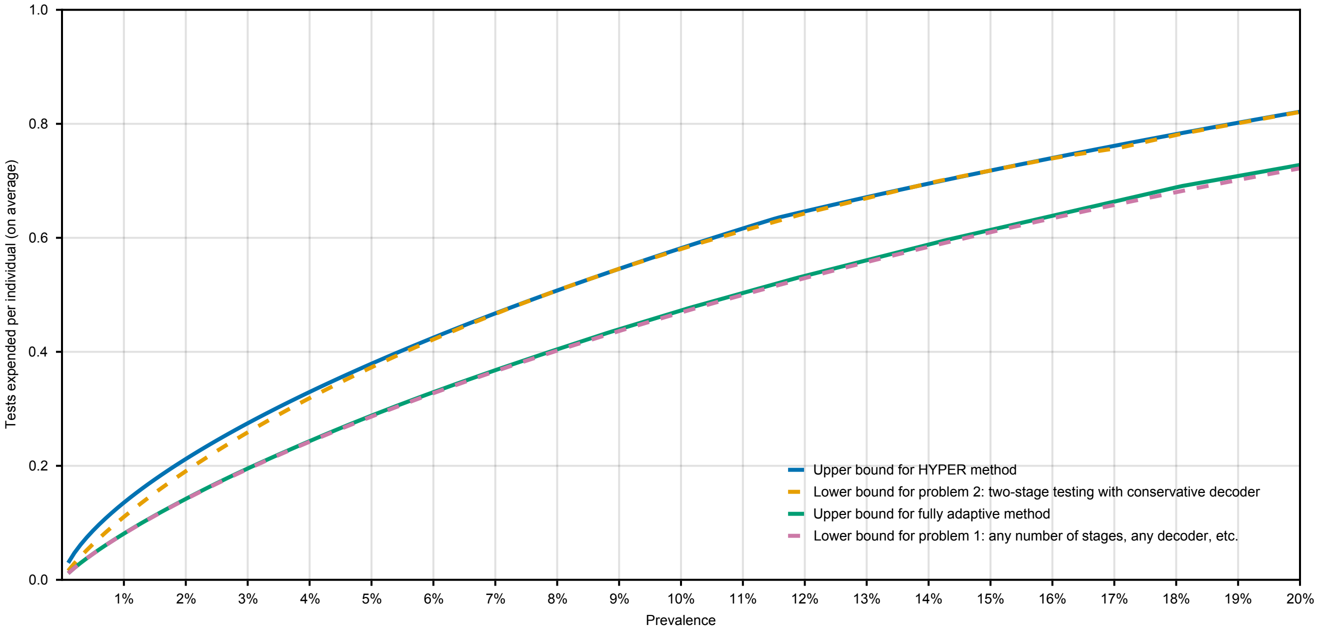

Supplementary Figure 2: Comparison of two design problems and their bounds. We compare two design problems. Problem 1 is to minimize the average number of tests used per individual by using any group testing method (i.e., any number of stages, any decoder, etc.). This problem is tackled, e.g., by fully-adaptive methods. Problem 2 is to minimize the average number of tests used per individual but by using two-stage group testing methods with a conservative decoder[32]. This is the problem that HYPER tackles. Namely, we consider methods with a first stage of pooled tests followed by a second stage where putative positives are tested individually. This class of methods is of great practical importance. Using only two stages reduces the time needed to receive results, which is crucial in public health settings. Moreover, carrying out more stages can be difficult in practice due to the added logistical burden (especially for adaptively chosen pooled tests). Using a conservative decoder also helps in practice since it avoids more complicated reasoning, e.g., to identify definite positives by process of elimination. This figure compares known lower bounds for the two problems in the noiseless large n setting (i.e., $\alpha = 0$, $\beta = 1$, and $n \to \infty$) for a range of fixed prevalences p (sometimes called the linear regime[33]). As one would naturally expect, the lower bound (counting bound[33]) for problem 1 is lower than the lower bound[32] for problem 2 since it is less constrained. For problem 1, fully-adaptive methods are nearly optimal, as was already known[33]. For problem 2, HYPER appears to be fairly close to optimal. The gap visible for smaller prevalences is likely due to: a) our additional constraint that $q \leq 3$ (to aid real-life implementation), b) looseness in our upper bound for HYPER, or c) potential looseness in the lower bound[32].

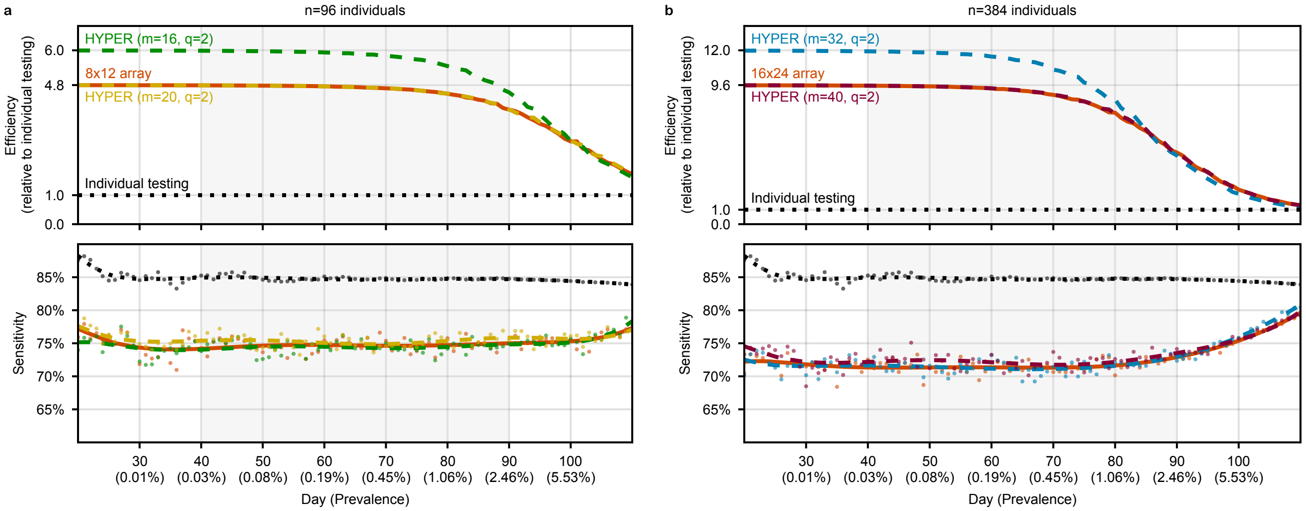

Supplementary Figure 3: Comparison of plate-based array designs with additional HYPER designs. We expand the comparison of HYPER and array designs in Fig. 2. Previously, the HYPER designs were chosen to have the same maximum pool sizes ($nq/m = 12$ for $\mathrm{H}_{96,16,2}$; $nq/m = 24$ for $\mathrm{H}_{384,16,2}$) as the array designs. Here we include HYPER designs that are instead chosen to have the same number of pools ($m = 20$ for the 8×12 array and $m = 40$ for the 16×24 array). These HYPER designs have efficiency nearly the same as their array counterparts. However, they have a slightly higher sensitivity, likely due to their more balanced pools.

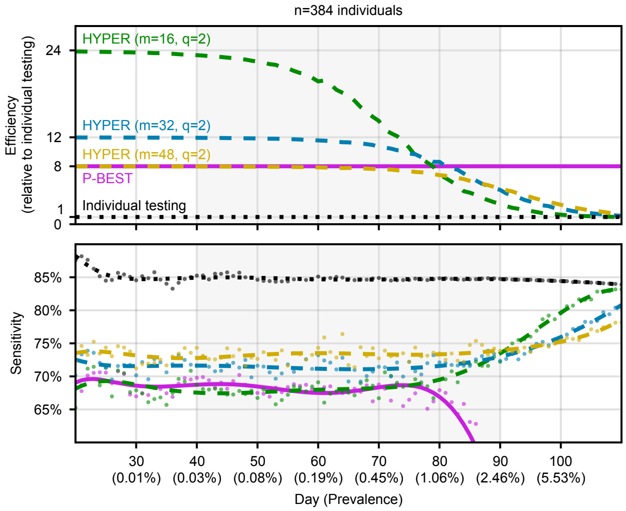

Supplementary Figure 4: Comparison of P-BEST with additional HYPER designs. We expand the comparison of HYPER and P-BEST in Fig. 2. To the previous $\mathrm{H}_{384,32,2}$ HYPER design, we add a $\mathrm{H}_{384,48,2}$ design (with the same number of pools $m = 48$ as P-BEST) and a $\mathrm{H}_{384,16,2}$ design (with same pool size $nq/m = 48$ as P-BEST). Since $\mathrm{H}_{384,48,2}$ has the same number of pools as P-BEST, its efficiency at low prevalence is similar. However, its pools are one-third the size helping it achieve a higher sensitivity. The $\mathrm{H}_{384,16,2}$ has the same size pools as P-BEST, and a comparable sensitivity for much of the 50-day window highlighted. However, it has one-third as many pools giving it an initial efficiency roughly three times higher. As before (Fig. 2), the efficiency of HYPER declines for both designs as prevalence grows, eventually falling below the constant efficiency achieved by P-BEST around day 80. Likewise, as before, the sensitivity of HYPER grows around the same time, while P-BEST significantly loses sensitivity.

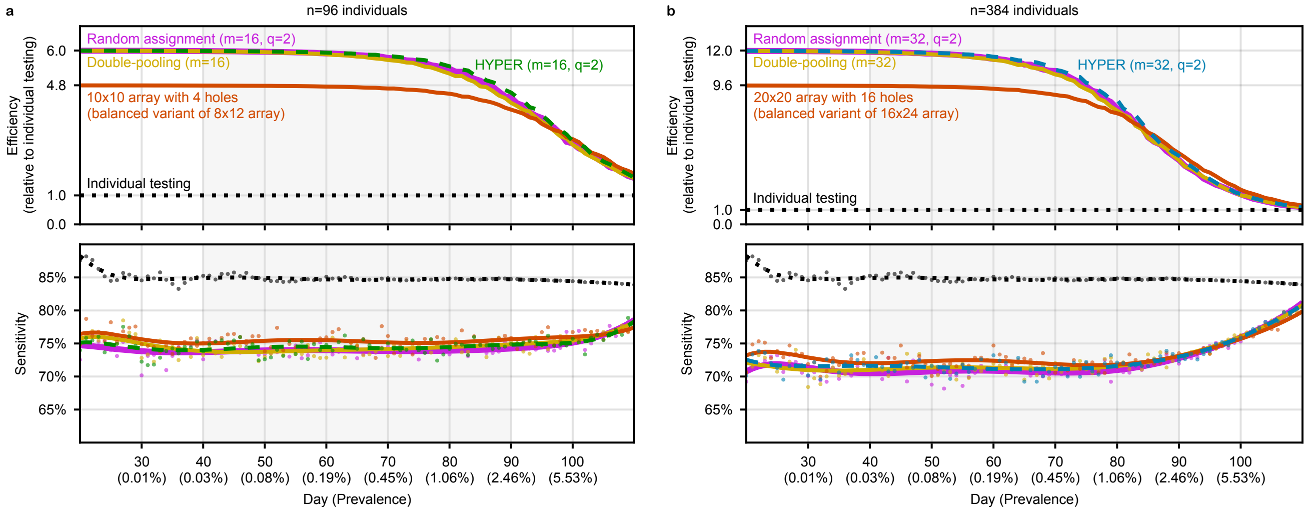

Supplementary Figure 5: Comparison with additional designs. We consider the simulated epidemic from Fig. 2, and compare HYPER (as in Figs. 2a and 2b) with balanced variants of the plate-based arrays (i.e., square arrays where some number of cells along the diagonal are empty), random assignment[1], and double-pooling[2]. As before, average values of efficiency (relative to individual testing) and sensitivity of the various pooling designs are shown for each day, with results averaged across 200,000 random trials. For sensitivity, raw averages are shown as dots with degree-8 polynomial fits overlaid as curves; the curves for efficiency depict raw averages. For both $n = 96$ (**a**) and $n = 384$ (**b**) individuals per batch, the average efficiency and sensitivity of both random assignment and double-pooling are generally similar to their corresponding HYPER designs. However, it turns out that these random designs behave less consistently than HYPER. Their performance depends on which individual happens to be positive, which is undesirable from a laboratory standpoint. This aspect is obscured by the averages in this figure, and we investigate it separately in Supplementary Fig. 7. Compared with the balanced variants of the plate-based arrays, HYPER uses fewer pools and is roughly 25% more efficient for much of the 50-day window highlighted. HYPER also has correspondingly larger pool sizes, and is slightly less sensitive. Note that the 10×10 and 20×20 arrays here are, respectively, the smallest square arrays that can accommodate $n = 96$ and $n = 384$ individuals without placing multiple individuals in the same array cell. Forming balanced arrays with the same number of pools as HYPER (i.e., $m = 8$ and $m = 16$) requires extending the design to assign multiple individuals to some array cells, and we consider these designs in Supplementary Fig. 6.

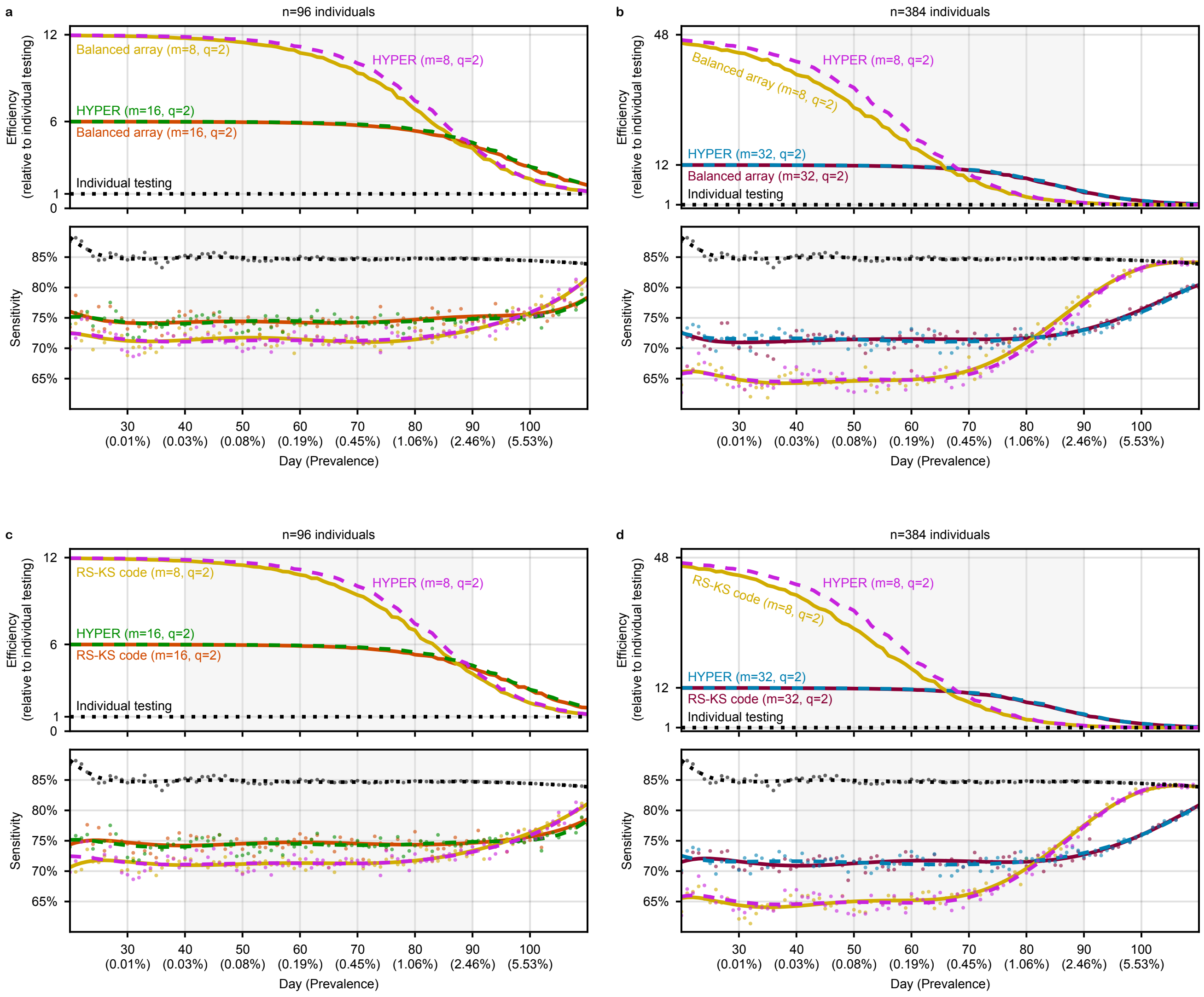

Supplementary Figure 6: Comparison with balanced array and code-based designs with matching parameters. Since plate-based arrays and P-BEST are particular instances of general array-based and code-based designs, we also compare HYPER with general balanced arrays (**a,b**) and Reed-Solomon Kautz-Singleton (RS-KS) code-based designs (**c,d**), with matching design parameters (number of individuals n, number of pools m, pools per individual q). We use the extended versions of these designs described in Sections S8 and S9; unextended versions do not accommodate more than $(m/2)^2$ individuals when $q = 2$. The sensitivity of these methods closely match those of HYPER when the pooling parameters are the same. Sensitivity depends significantly on the number of individuals per pool, which is nq/m for all three methods since they all have balanced pools. HYPER has either similar or better efficiency than both methods, with a larger improvement arising for more aggressive pooling parameters that use fewer pools and are more efficient at low prevalence.

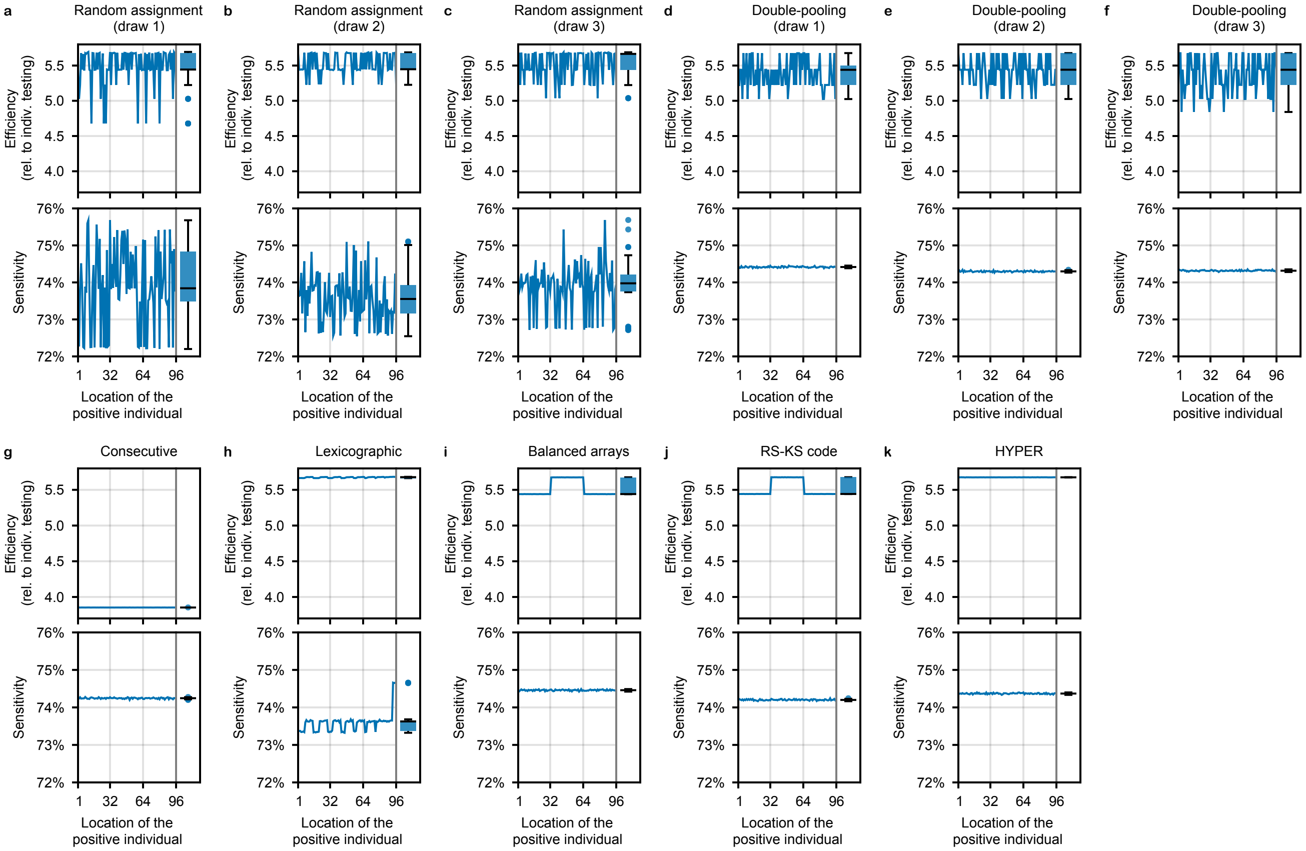

Supplementary Figure 7: Impact of imbalanced designs. We investigate how balance affects how much efficiency and sensitivity vary depending on where positive individuals happen to fall. We consider designs with $n = 96$ individuals per batch, $m = 16$ pools and $q = 2$ splits, and we suppose exactly one individual is positive in each batch. That individual's viral load is drawn from the distribution of nonzero viral loads on day 80 of the simulated epidemic from Fig. 2 (for which the prevalence is roughly 1.06%); everyone else has zero viral load. We consider placing the positive individual in each of the n slots of the batch (i.e., as individual 1, individual 2, etc.), and compare the average values (from 100,000 trials) for efficiency (relative to individual testing, i.e., individuals/test) and for sensitivity as functions of the location. Random assignment[1] (**a-c**) has varying performance depending on which particular design gets drawn, and often has unbalanced pool combinations and pools. As a result, all three draws had uneven efficiency and sensitivity; e.g., for the first draw, the sensitivity was 75.7% for a positive individual in location 11 but 72.2% for location 7. Double-pooling[2] (**d-f**) guarantees balanced pools but often has unbalanced pool combinations. As a result, all three draws had uniform sensitivity but uneven efficiency. Note that uneven efficiency means that the stage 2 workload is more unpredictable, which can make the logistics of large-scale screening more difficult. Consecutive pooling (AB, CD, EF, ...; **g**), only uses a subset of the $\binom{m}{q}$ possible pool combinations and was generally less efficient. It does, however, have balanced pools yielding uniform sensitivity regardless of where the positive individual fell. Lexicographic pooling (AB, AC, AD, ...; **h**) has balanced pool combinations yielding uniformly high efficiency. However, it has unbalanced pools resulting in uneven sensitivity. Balanced array pooling (**i**) and Reed-Solomon Kautz-Singleton (RS-KS) code-based pooling (**j**) both had balanced pools here, and each was maximally balanced on the $(m/2)^2$ pool combinations it used. However, neither was perfectly balanced on the pool combinations it used; in both cases, some pool combinations were used twice while others were used once. Both had uniform sensitivity and slightly uneven efficiency. In contrast, HYPER (**k**) has both balanced pool combinations and balanced pools. As a result, it had both uniformly high efficiency and uniform sensitivity. Moreover, its median efficiency (5.68 individuals/test) and median sensitivity (74.4%) were generally among the best. The boxplots include a center line (median), box limits (upper and lower quartiles), whiskers (1.5x the interquartile range), and points (outliers).

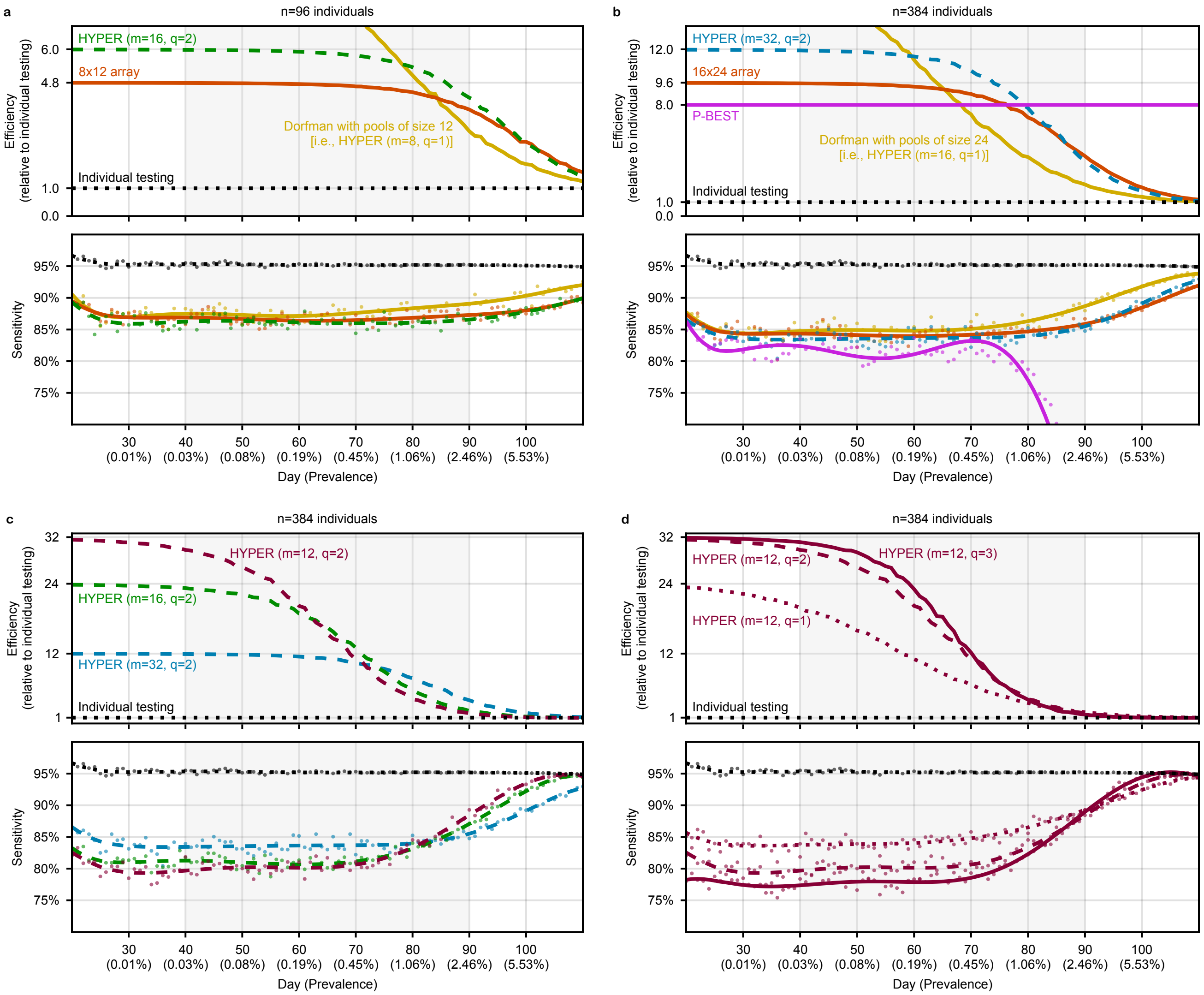

Supplementary Figure 8: Efficiency and sensitivity of pooled testing during a simulated epidemic (Fig. 2) repeated for a 25-fold reduction in the limit of detection (from 100 to 4). With this reduction in the limit of detection (LOD), smaller viral loads are detected resulting in a higher individual testing sensitivity of roughly 95%. The sensitivity of the group testing methods increased concordantly, resulting in similar relative sensitivities as before. For example, the $H_{96,16,2}$ design and the 8×12 array design have a 0.54 percentage point difference in sensitivity averaged across days 40-90 here, compared with a 0.24 percentage point difference before (Fig. 2). The sensitivity loss with respect to individual testing is now slightly smaller; e.g., the sensitivity of the $H_{96,16,2}$ design is roughly 9 percentage points lower here, compared with a roughly 10 percentage point difference before (Fig. 2). In general, an increase in sensitivity can lead to a decrease in efficiency, since more pools (correctly) test positive, but the impact appears to be small here. Efficiency for the various methods is very similar to before (Fig. 2), with HYPER enjoying essentially the same gains in efficiency over individual testing.

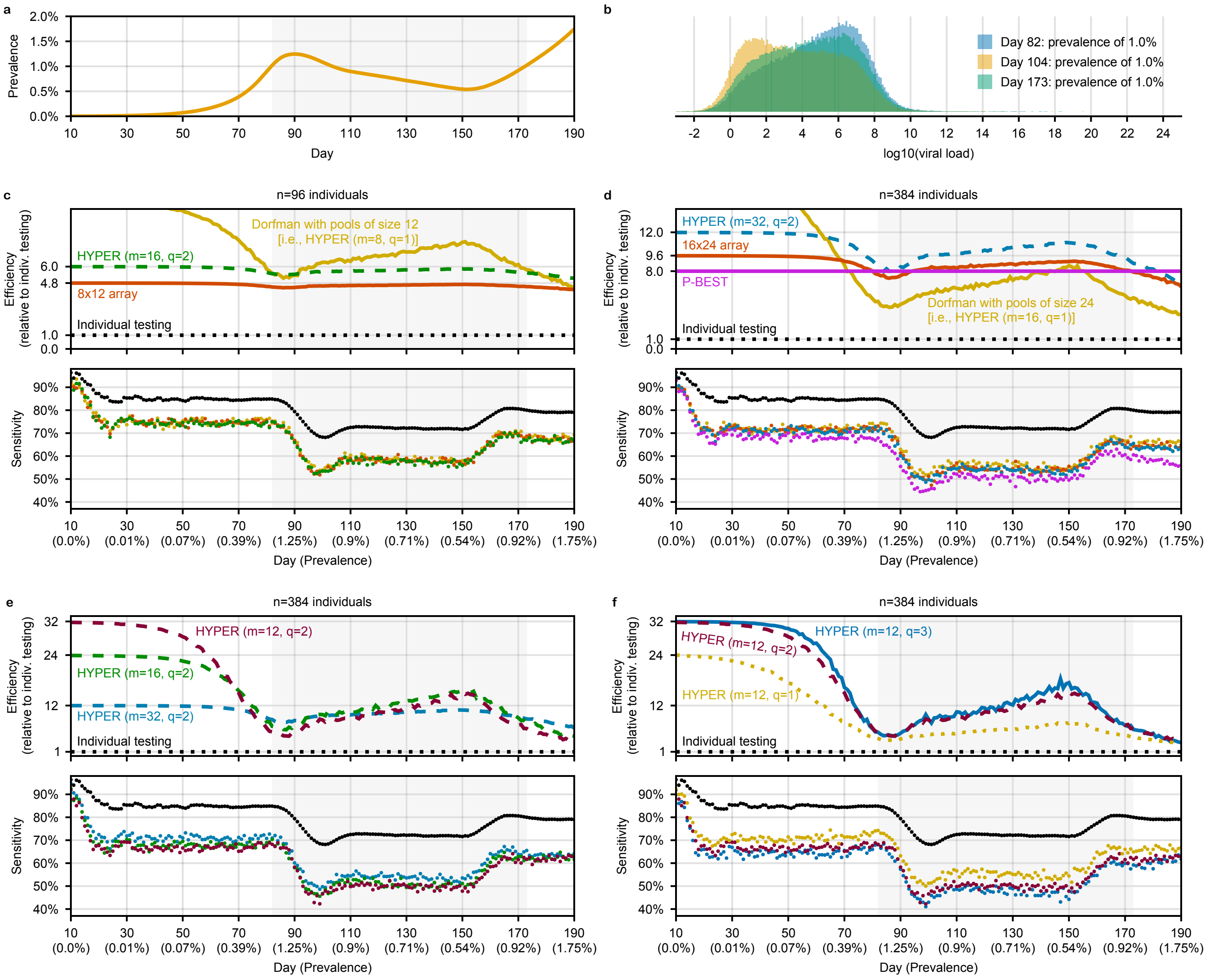

Supplementary Figure 9: Efficiency and sensitivity of pooled testing during a simulated epidemic (Fig. 2) repeated with a two-wave epidemic. To evaluate a later (post-exponential) phase, we repeated our simulation but with a population undergoing a sustained, two-wave epidemic (obtained from Cleary and Hay et al.[1]). The simulated population was generated from an SEIR model with transmission rate modified at two time points: R_0 was initiated at 2.5 at day 0, decreased to 0.8 at day 80, and subsequently increased to 1.5 at day 150. The result is an epidemic with an initial wave, followed by a decline phase and subsequently another growth phase (**a**). We compare the efficiency and sensitivity of the same methods as before (**c,d,e,f**). Consistent with earlier studies[1], sensitivity is generally lower for all methods (including individual testing) during the decline phase compared to the two growth phases. For example, compare day 104 (during decline) with days 82 and 173 (during growth), which all had prevalence around 1.0%. The difference in sensitivity can be explained by observing that the distribution of nonzero viral loads (**b**) on day 104 is shifted to the left relative to days 82 and 173, due to a shift away from recent infections. Hence, positive viral loads are more likely to fall below the limit of detection and get missed. At the same time, the relative performance of the methods is similar to before. The chosen HYPER designs are as sensitive as their corresponding array designs, while being roughly 20-25% more efficient for $n = 96$ and roughly 10-25% more efficient for $n = 384$. For $n = 384$, the HYPER design is generally more efficient than P-BEST (up to 50% more) until day 180 while also being generally more sensitive. After day 180, P-BEST is more efficient but at an additional cost of sensitivity.

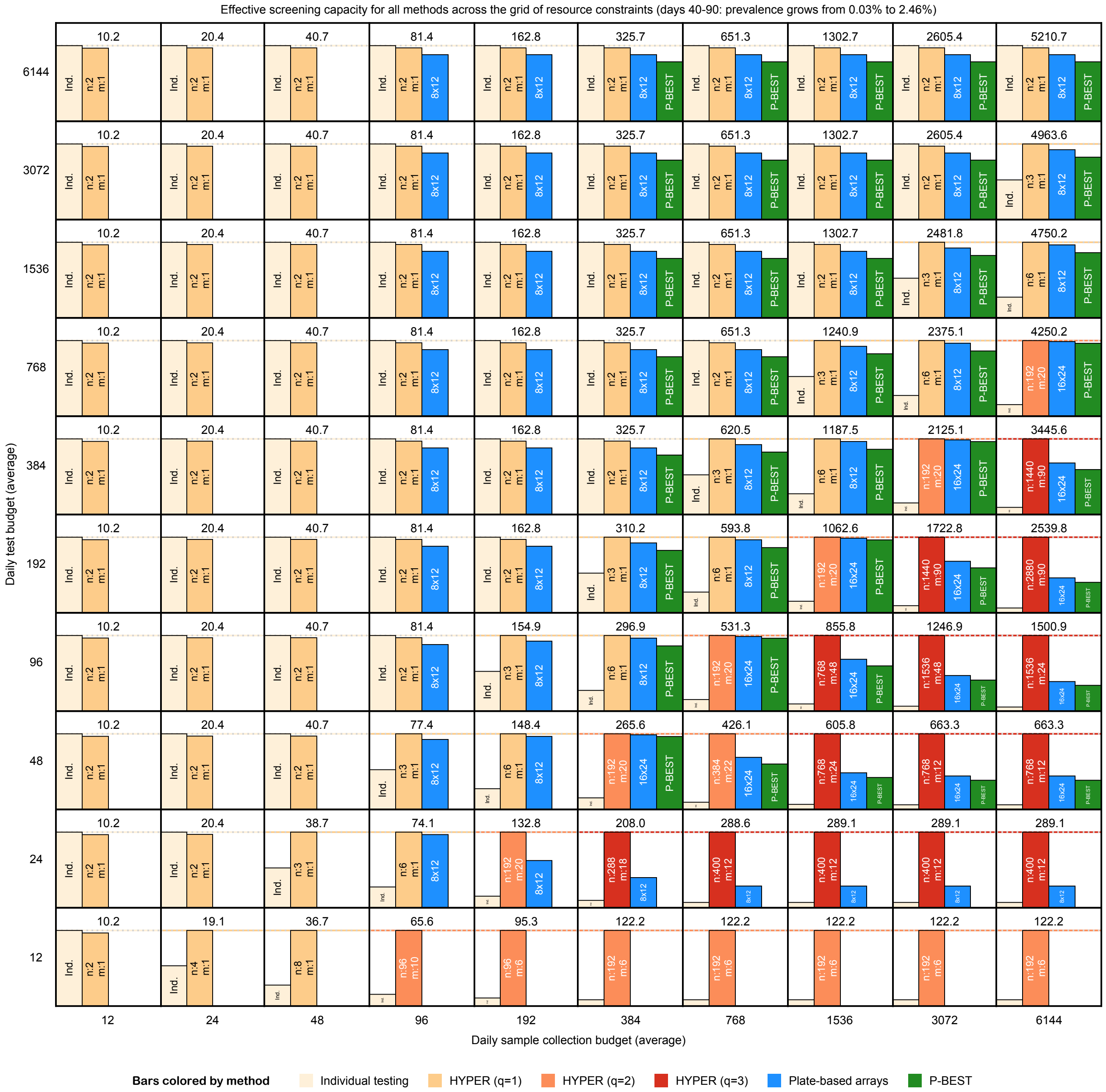

Supplementary Figure 10: Detailed comparison corresponding to Fig. 3. Each cell shows the effective screening capacity given constrained sample collection and testing budgets, for individual testing, HYPER (best n and m in annotation with q indicated by bar color), plate-based arrays[4] (better design between 8×12 and 16×24 noted in white), and P-BEST[3]. The best effective screening capacity for each cell is shown in black.

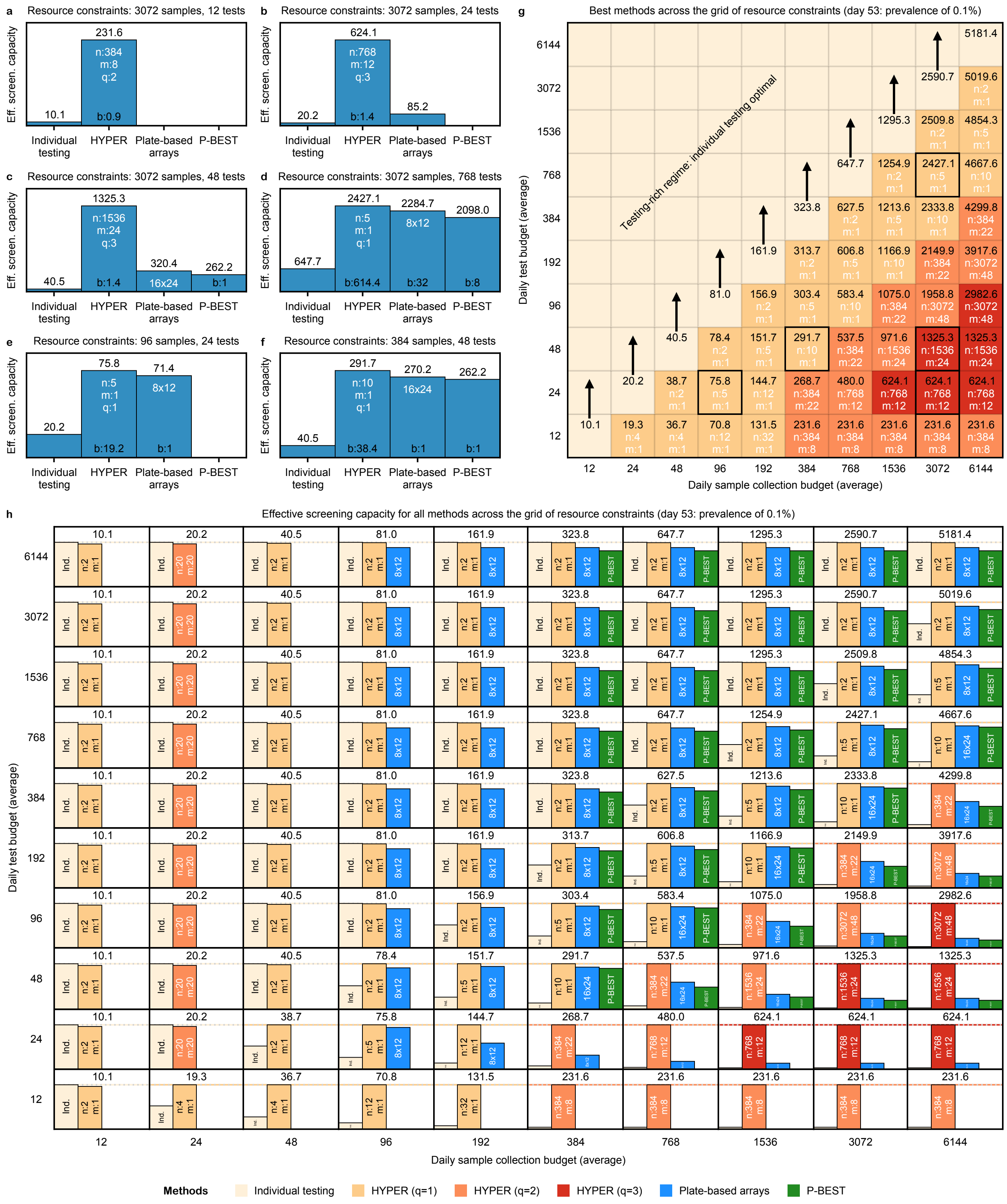

Supplementary Figure 11: Comparison of pooling methods under resource constraints (Fig. 3 and Supplementary Fig. 10) repeated for day 53 (prevalence of 0.1%). At this low prevalence, HYPER is the most effective across the grid of resource constraints and can be significantly so. The low prevalence setting also favors simple designs (low q) across more of the test-constrained regime.

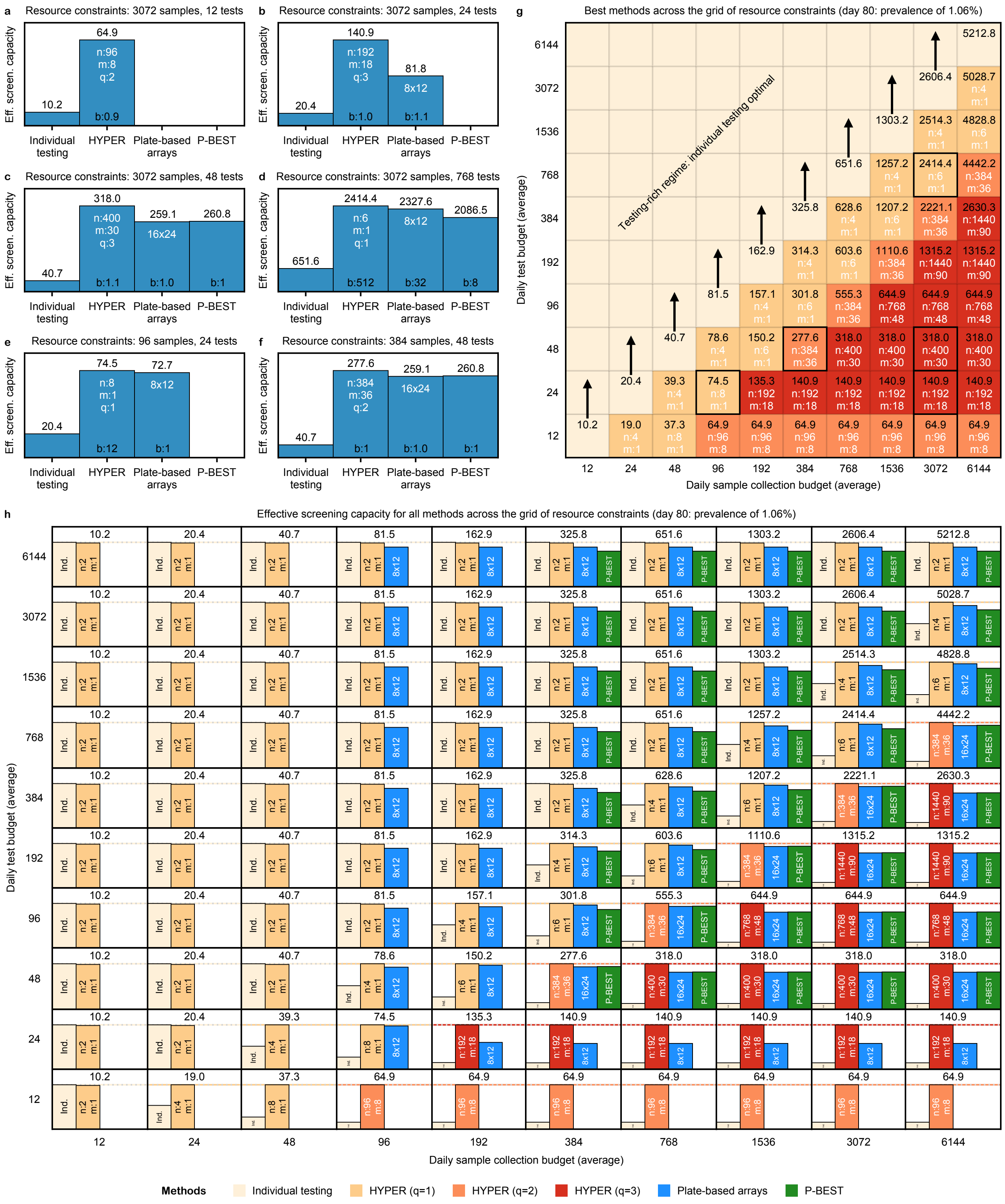

Supplementary Figure 12: Comparison of pooling methods under resource constraints (Fig. 3 and Supplementary Fig. 10) repeated for day 80 (prevalence of 1.06%). At this moderate prevalence, HYPER remains best across all scenarios. Compared to Supplementary Fig. 11, which had lower prevalence, designs with higher q are more often the most effective.

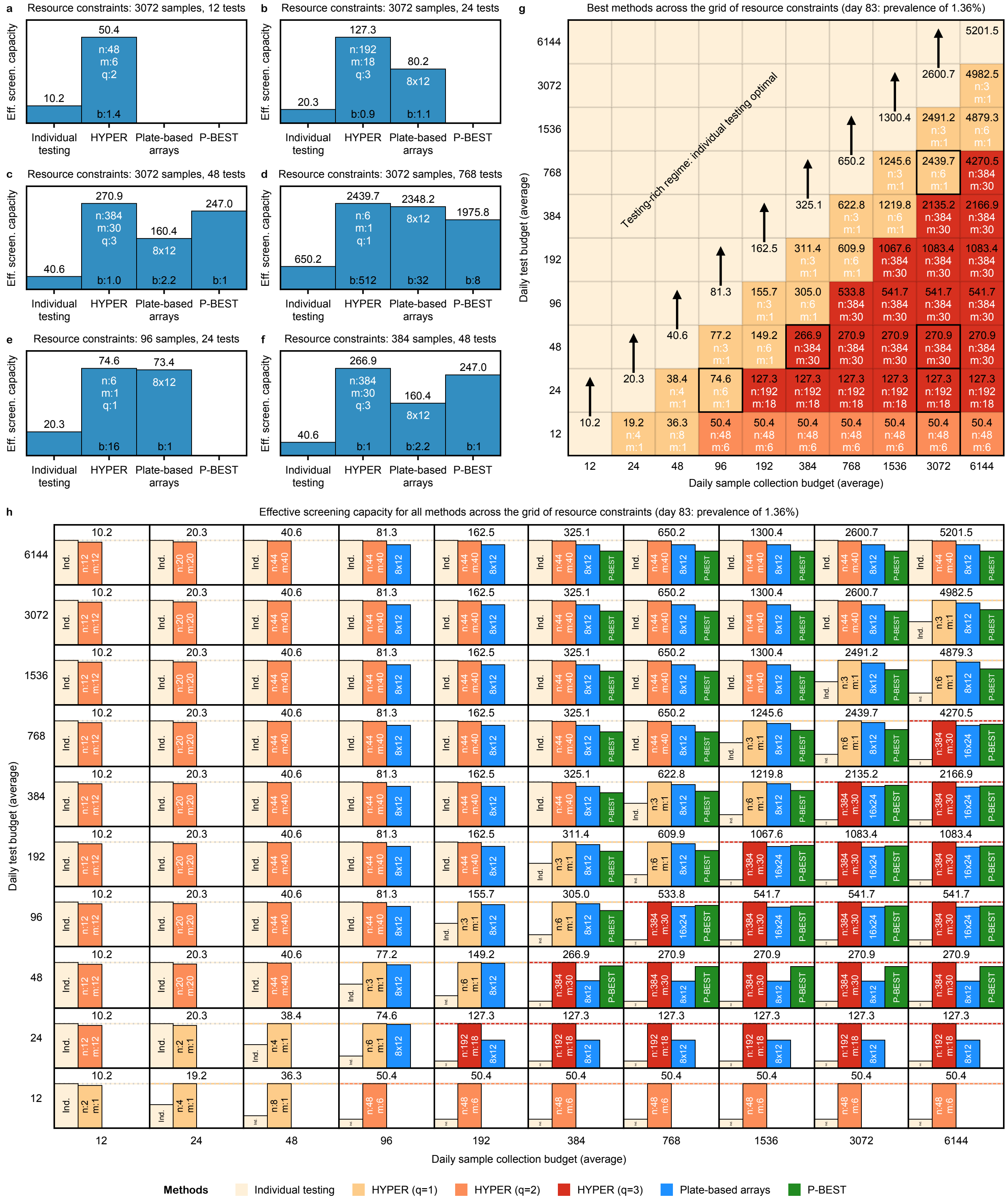

Supplementary Figure 13: Comparison of pooling methods under resource constraints (Fig. 3 and Supplementary Fig. 10) repeated for day 83 (prevalence of 1.36%). At this prevalence, HYPER remains best across all scenarios. Compared to Supplementary Fig. 12, designs with higher q are even more often the most effective.

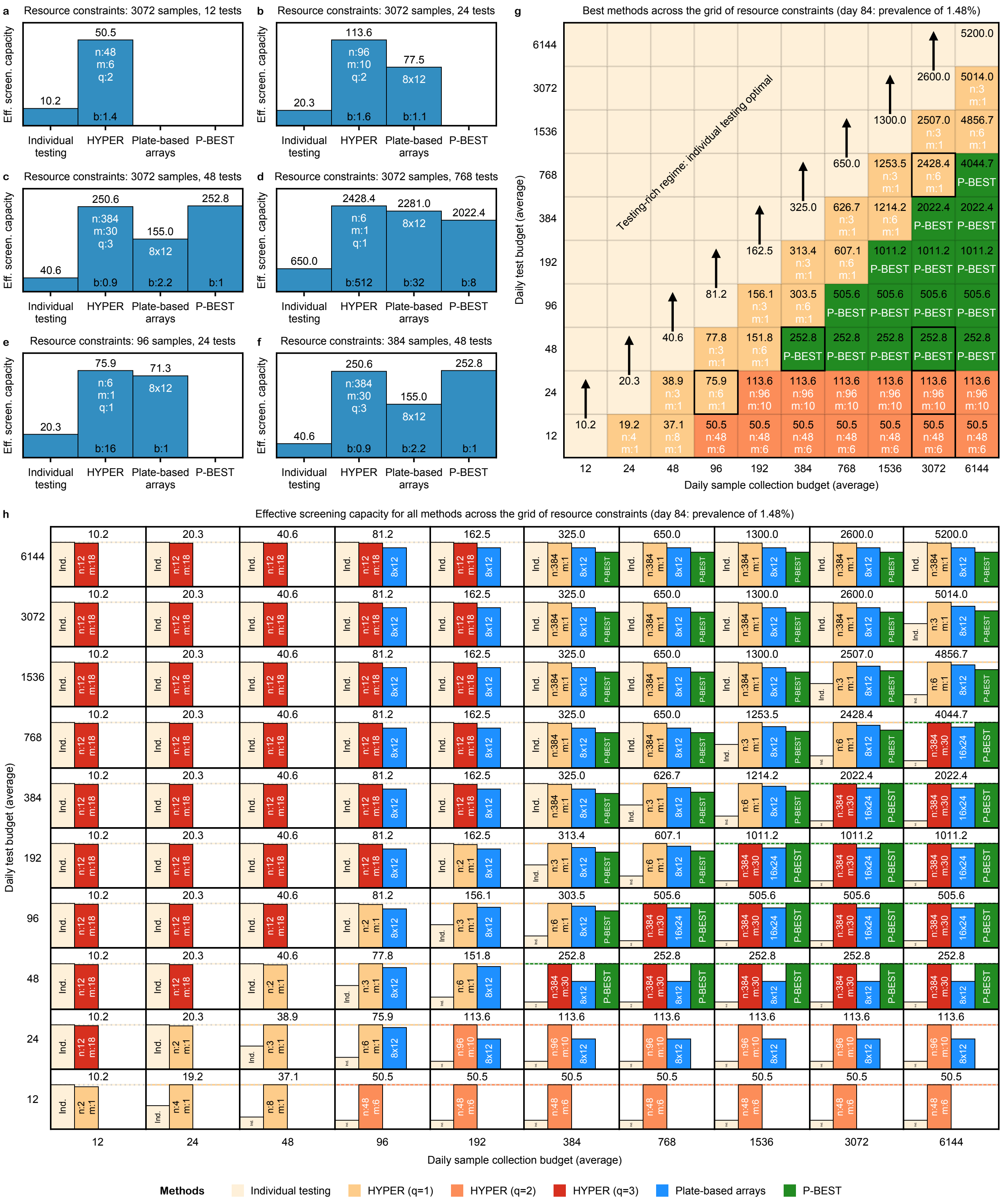

Supplementary Figure 14: Comparison of pooling methods under resource constraints (Fig. 3 and Supplementary Fig. 10) repeated for day 84 (prevalence of 1.48%). At this intermediate prevalence, there is a subset of scenarios in which P-BEST outperforms HYPER. However, HYPER is still nearly as effective in these scenarios. Outside these settings P-BEST is either not viable or substantially under-performs HYPER.

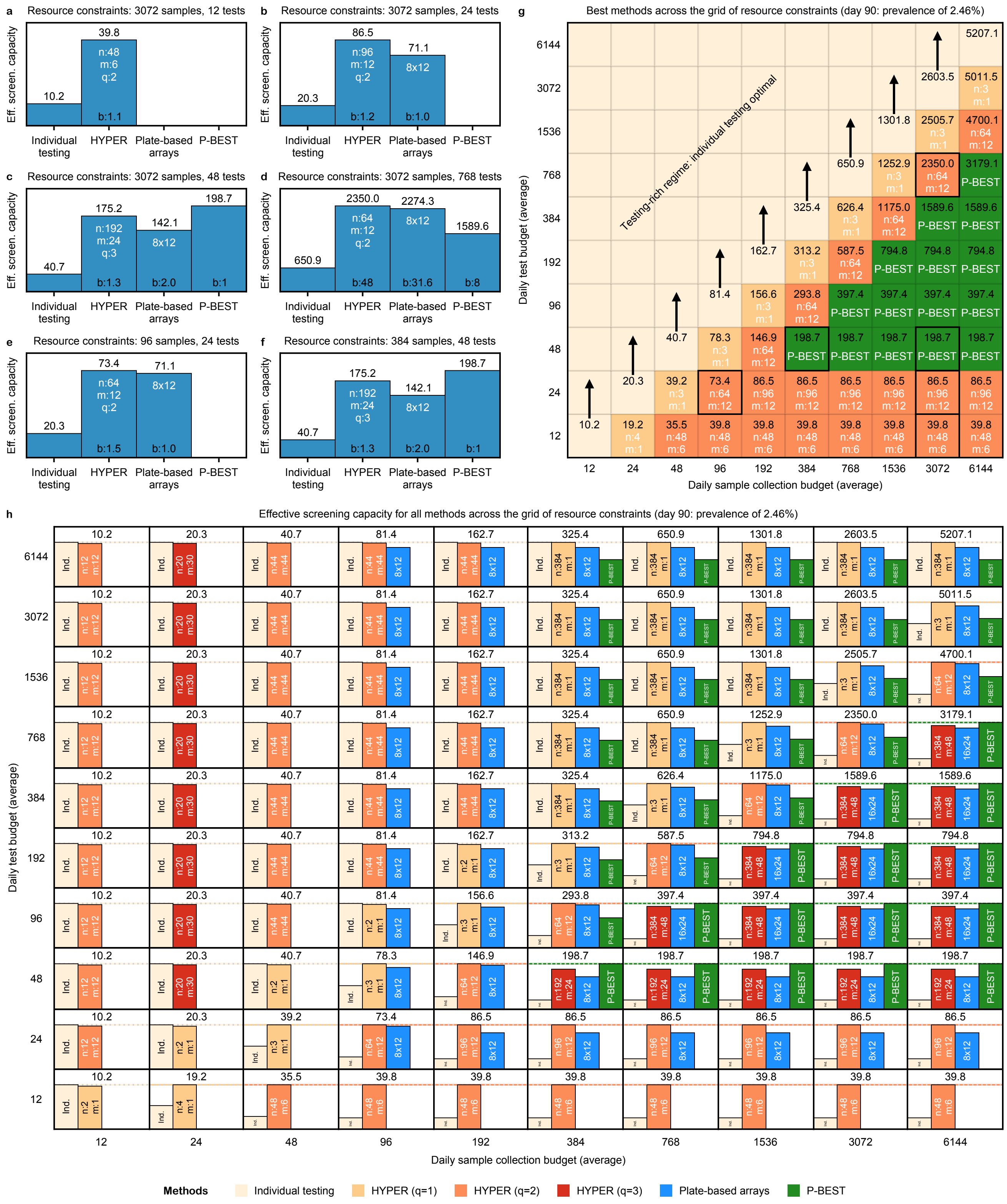

Supplementary Figure 15: Comparison of pooling methods under resource constraints (Fig. 3 and Supplementary Fig. 10) repeated for day 90 (prevalence of 2.46%). At this prevalence, P-BEST continues to outperform HYPER on a subset of scenarios, with a larger gap than in Supplementary Fig. 14. Outside these settings P-BEST is still either not viable or substantially under-performs HYPER.

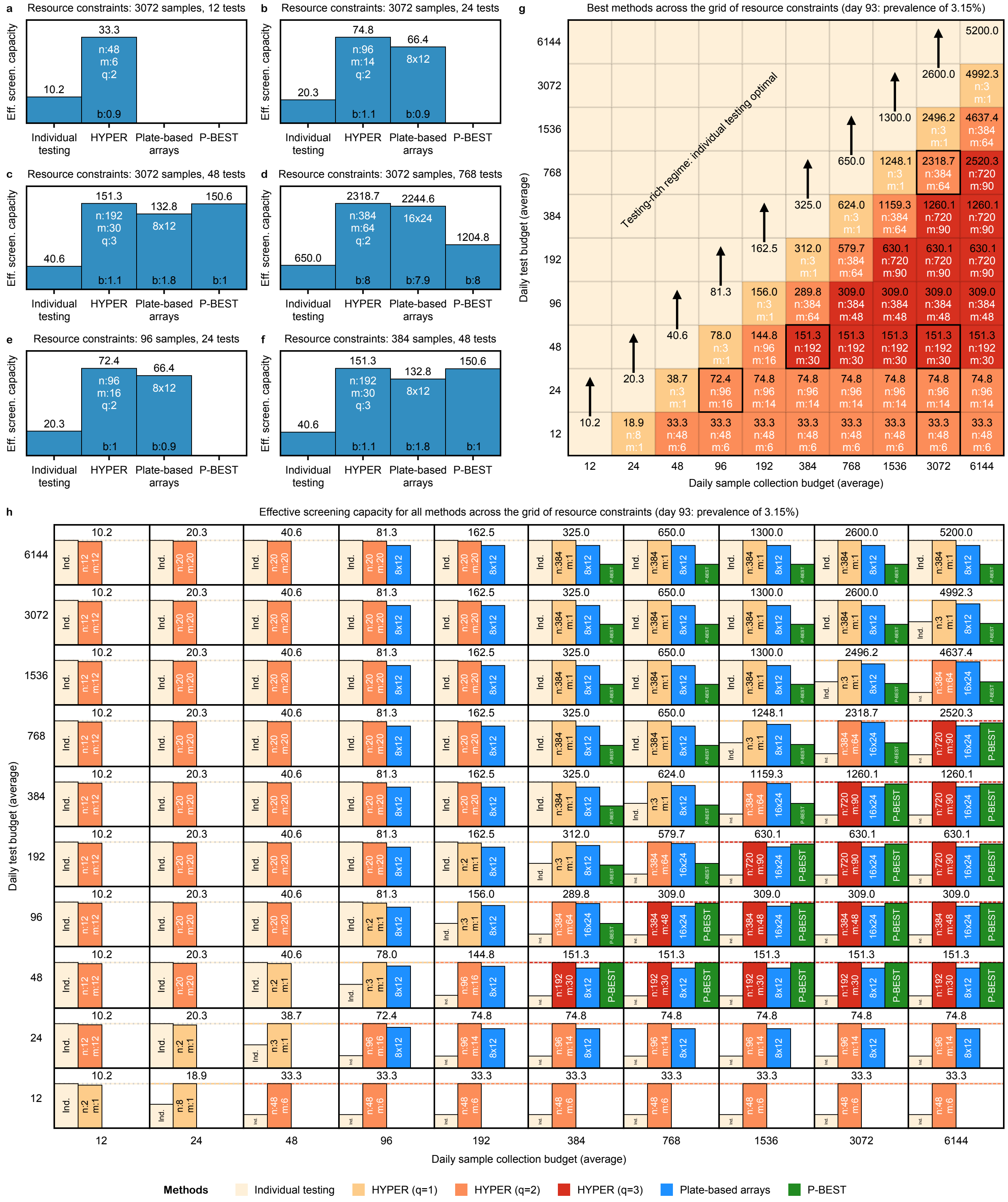

Supplementary Figure 16: Comparison of pooling methods under resource constraints (Fig. 3 and Supplementary Fig. 10) repeated for day 93 (prevalence of 3.15%). At this higher prevalence, HYPER again performs best across all scenarios.

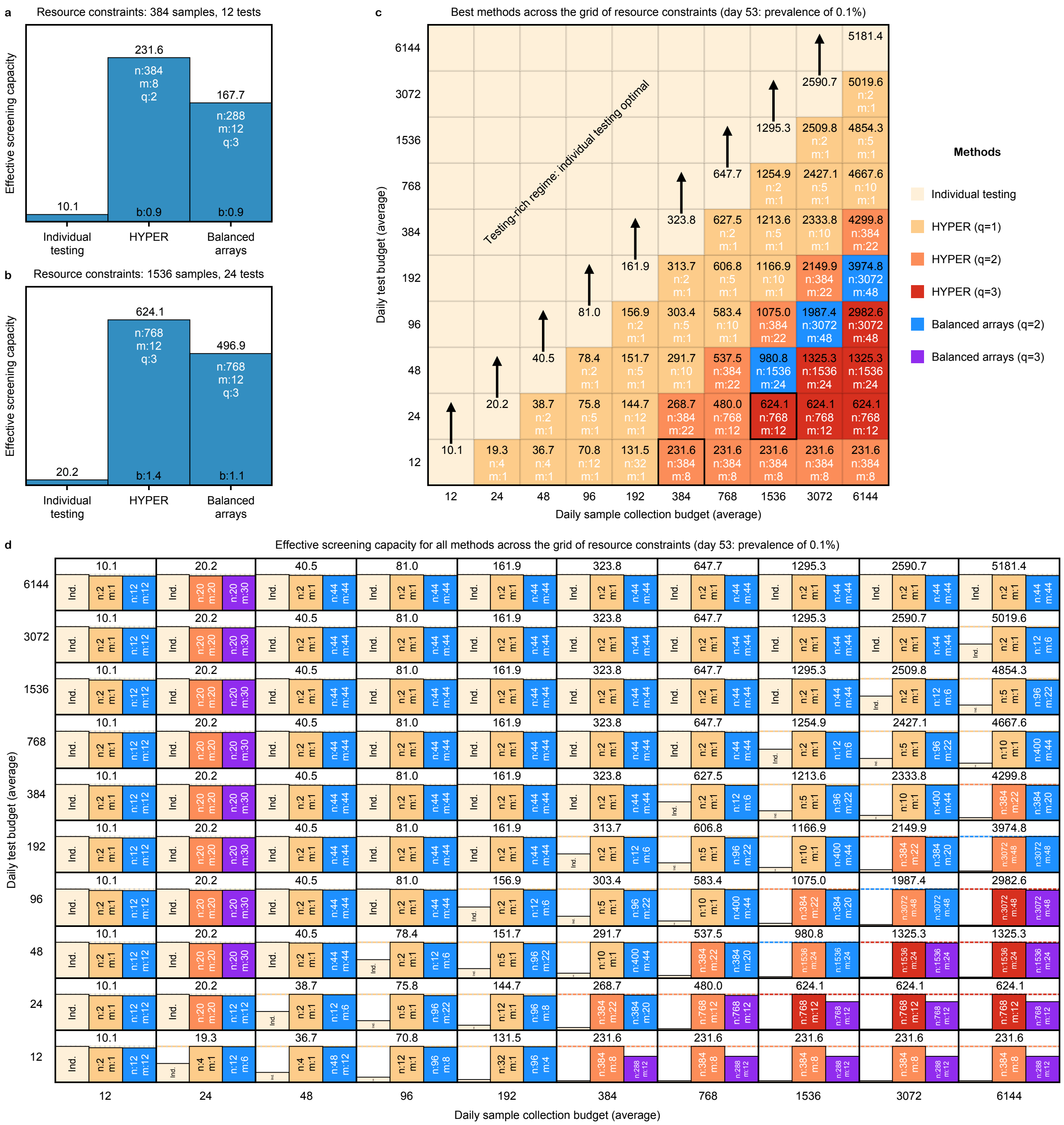

Supplementary Figure 17: Comparison with balanced array designs under resource constraints for day 53 (prevalence of 0.1%). HYPER was about as effective as or substantially more effective than balanced arrays across the entire grid of resource constraints (**c**, **d**). Both methods were optimized over the same sweep of n, m and q (Table 2). In important testing-constrained scenarios (with sample collection budget significantly outstripping testing budget), HYPER was up to 38% more effective than balanced arrays (e.g., for a budget of 384 samples and 12 tests; **a**). In some cases, HYPER was more effective because its greater efficiency enabled it to use more aggressive design parameters (**a**). In some other cases, the best design parameters for both methods was the same, but HYPER was more effective with the same parameters (e.g., for a budget of 1536 samples and 24 tests; **b**).

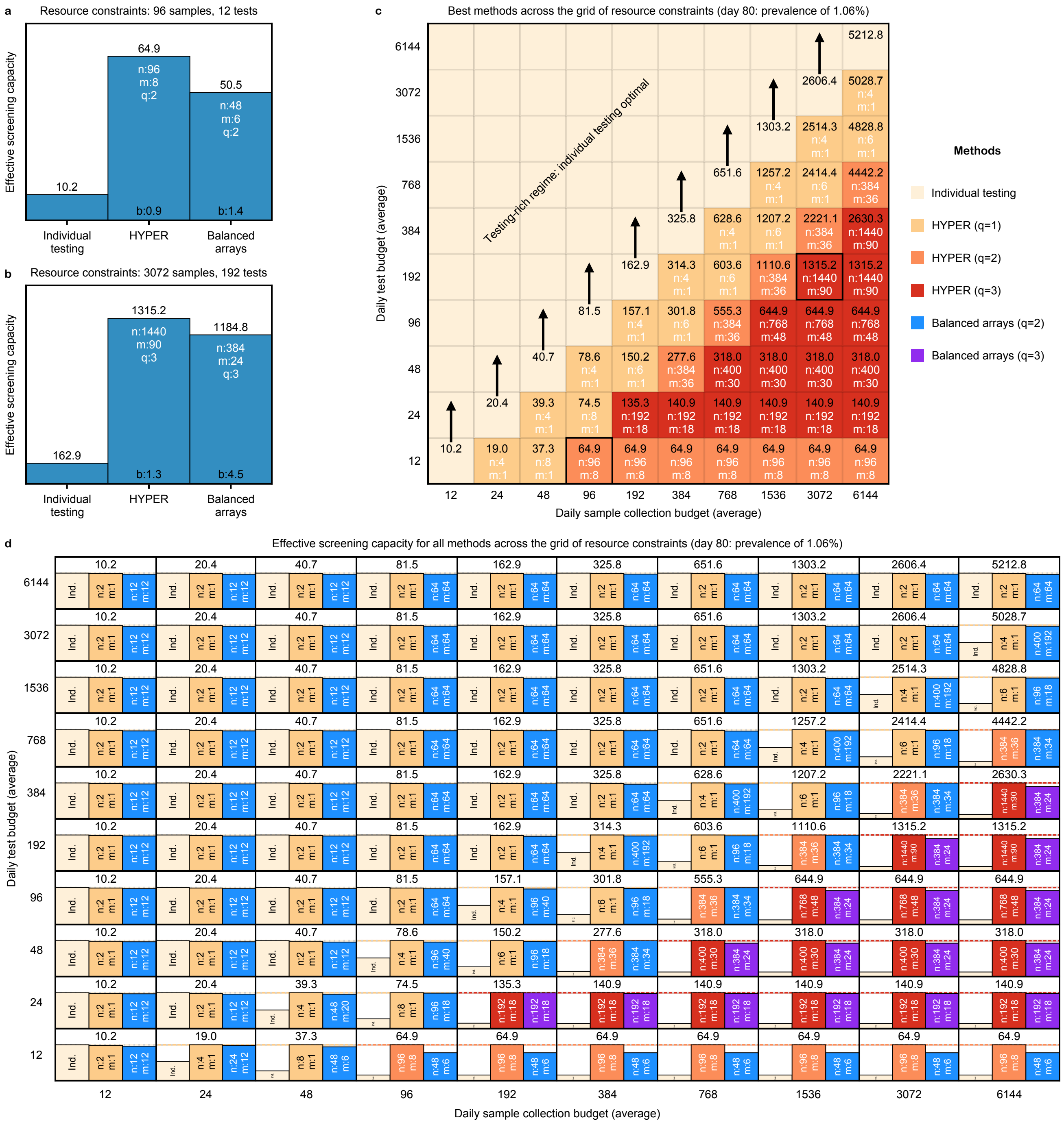

Supplementary Figure 18: Comparison with balanced array designs under resource constraints for day 80 (prevalence of 1.06%). We repeated the comparison of Supplementary Fig. 17 for a later day with higher prevalence. As before, HYPER was more effective than balanced arrays across the entire grid of resource constraints (**c**, **d**), where both were optimized over the same set of design parameters (Table 2). HYPER was also again significantly more effective than balanced arrays in important testing-constrained scenarios (with sample collection budget significantly outstripping testing budget). For example, HYPER was around 28% more effective than balanced arrays for a daily budget of 96 samples and 12 tests (**a**) and around 11% more effective for a daily budget of 3072 samples and 192 tests (**b**).

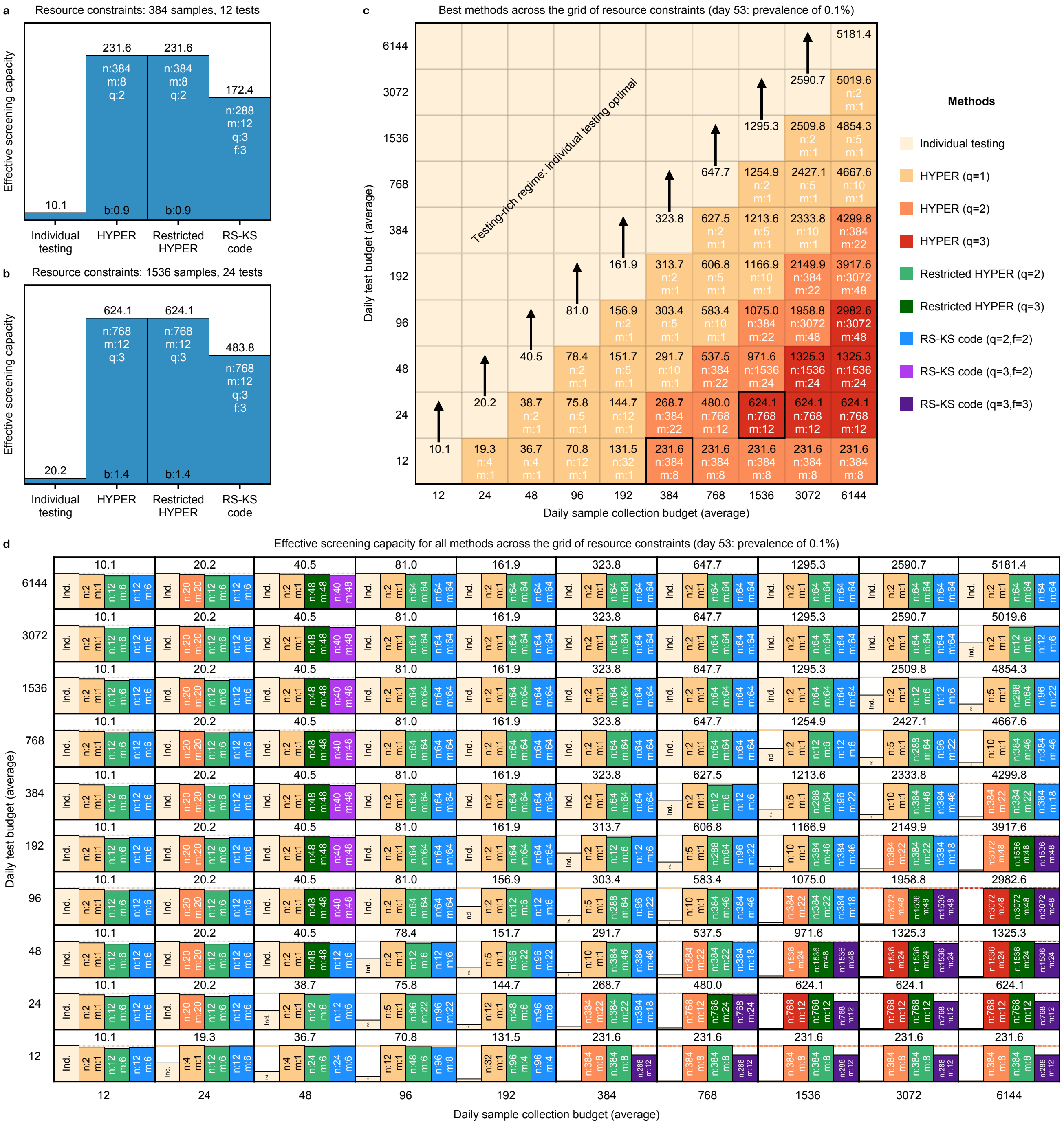

Supplementary Figure 19: Comparison with Reed-Solomon Kautz-Singleton (RS-KS) code-based designs under resource constraints for day 53 (prevalence of 0.1%). HYPER was about as effective as or substantially more effective than RS-KS across the entire grid of resource constraints (**c**, **d**), even when restricted to only design parameters allowed by RS-KS (Table 2 gives both the set used by HYPER and the restricted set used by Restricted HYPER and RS-KS). In important testing-constrained scenarios (with sample collection budget significantly outstripping testing budget), both HYPER and Restricted HYPER were up to 34% more effective than RS-KS (e.g., for a budget of 384 samples and 12 tests; **a**). In some cases, they were more effective because the greater efficiency of HYPER enabled them to use more aggressive design parameters (**a**). In some other cases, the best design parameters for all methods was the same, but HYPER and Restricted HYPER were more effective with the same parameters (e.g., for a budget of 1536 samples and 24 tests; **b**).

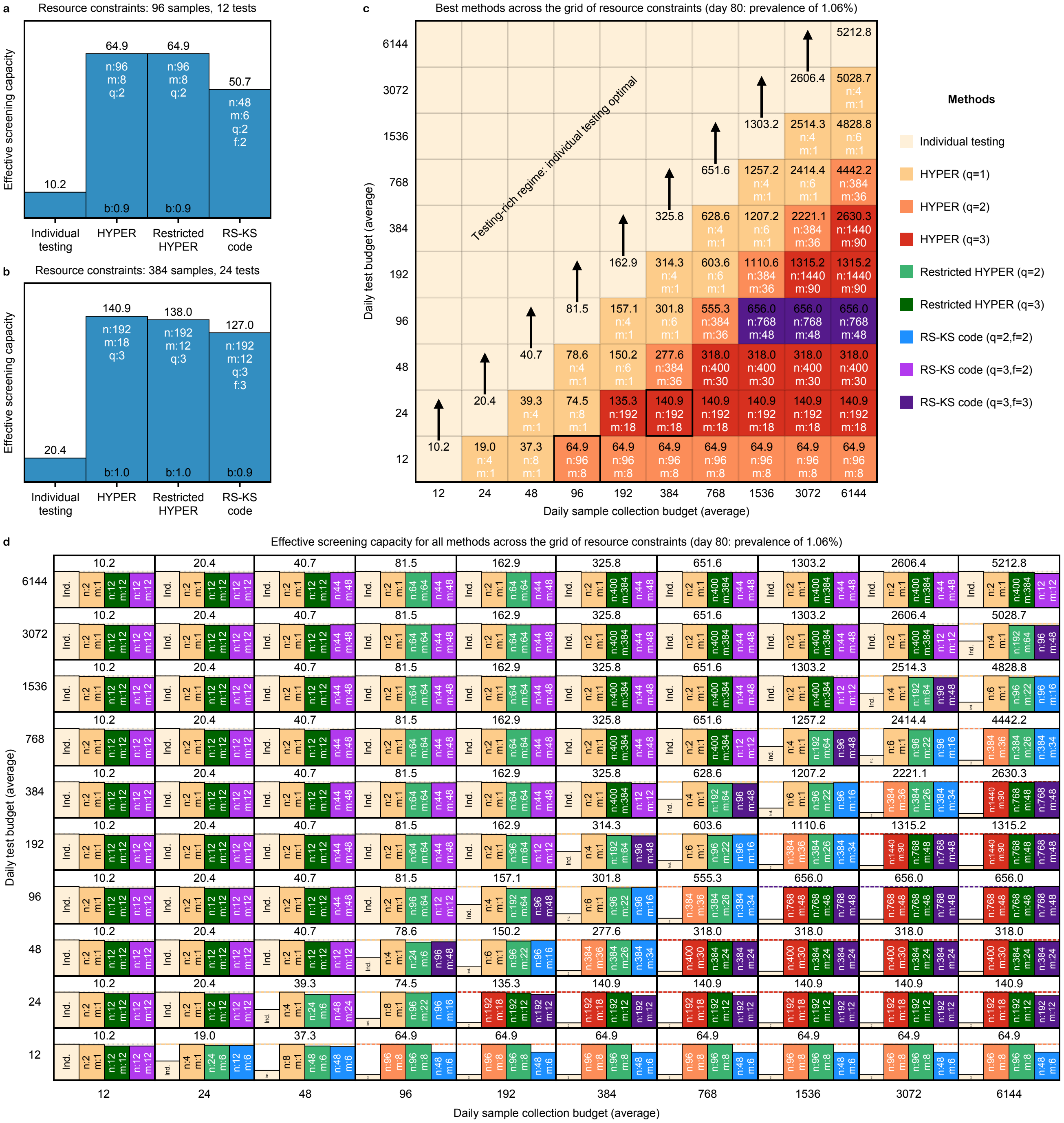

Supplementary Figure 20: Comparison with Reed-Solomon Kautz-Singleton (RS-KS) code-based designs under resource constraints for day 80 (prevalence of 1.06%). We repeated the comparison of Supplementary Fig. 19 for a later day with higher prevalence. As before, HYPER and Restricted HYPER were more effective than RS-KS across the entire grid of resource constraints (**c**, **d**), where Restricted HYPER was optimized over the same set of design parameters as RS-KS (Table 2). HYPER and Restricted HYPER were also again significantly more effective than RS-KS in important testing-constrained scenarios (with sample collection budget significantly outstripping testing budget). For example, both were around 28% more effective for a daily budget of 96 samples and 12 tests (**a**) and over 8% more effective for a daily budget of 384 samples and 24 tests (**b**).

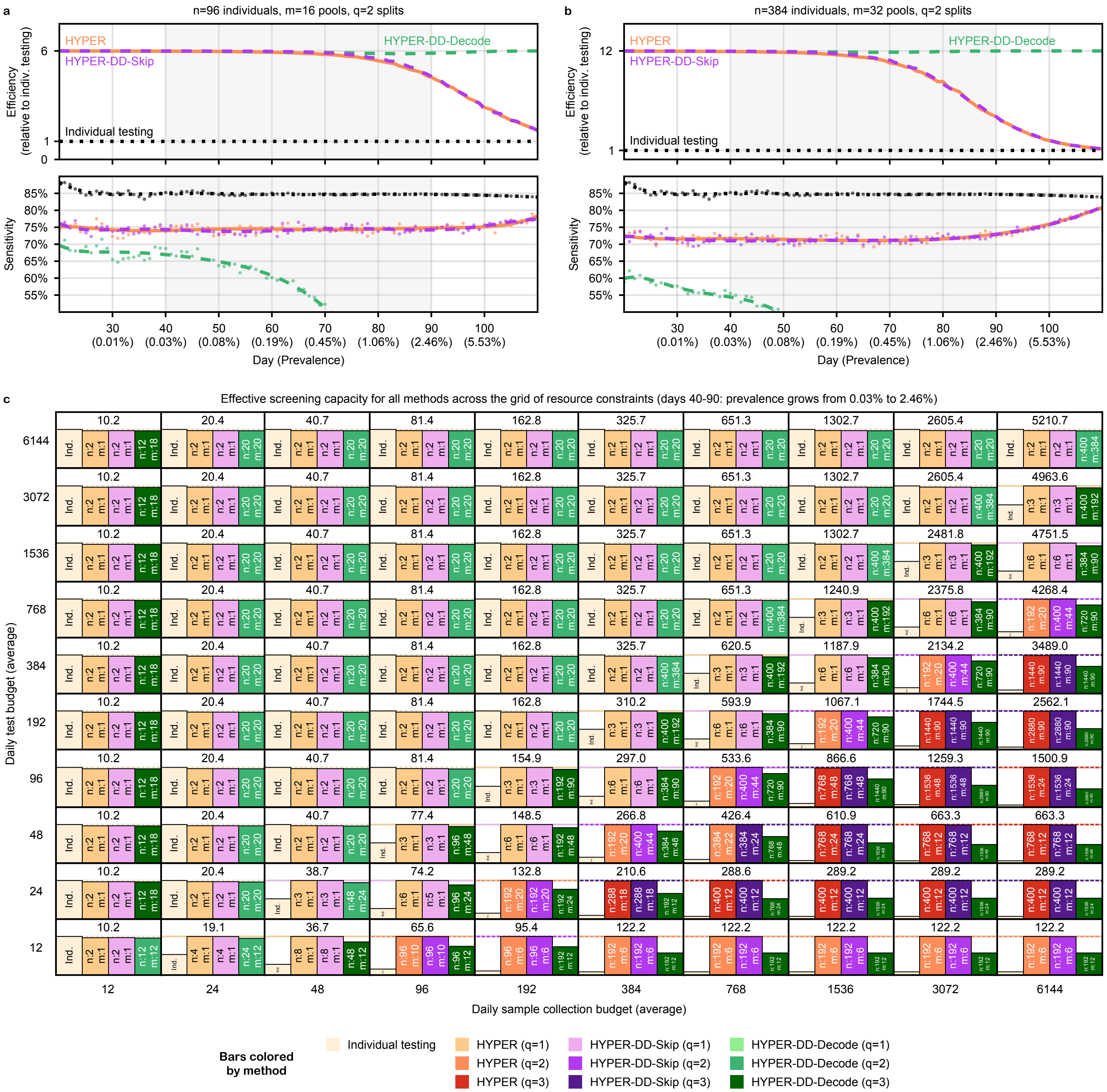

Supplementary Figure 21: Definite defective (DD) decoding. DD decoding[34;35] selects individuals who are the only putative positive in a positive test. We compare HYPER with two DD variants: 1) HYPER-DD-Decode replaces conservative decoding in HYPER with DD decoding, 2) HYPER-DD-Skip uses conservative decoding but skips stage 2 testing for DD individuals and declares them positive. **a,b** Average values of efficiency and sensitivity for each day (as in Fig. 2). For both designs considered ($H_{96,16,2}$ and $H_{384,32,2}$), HYPER-DD-Decode has nearly constant efficiency across time, but loses significant sensitivity as the prevalence grows. HYPER-DD-Skip has very similar efficiency and sensitivity as HYPER. **c** Effective screening capacity under resource constraints (as in Fig. 3 and Supplementary Fig. 10). HYPER-DD-Skip has a very similar effective screening capacity as HYPER, whereas HYPER-DD-Decode is substantially less effective in testing-constrained settings. Overall, both DD variants have the potential for higher efficiency (since they test a subset of the putative positives in stage 2), but neither significantly improves upon HYPER here. HYPER-DD-Decode sacrifices substantial sensitivity (since it declares non-DD individuals negative), and HYPER-DD-Skip does not gain much efficiency.

References

[1] Cleary, B. *et al.* Using viral load and epidemic dynamics to optimize pooled testing in resource-constrained settings. *Science Translational Medicine* **13** (2021).

[2] Broder, A. Z. & Kumar, R. A note on double pooling tests. Preprint at `http://arxiv.org/abs/2004.01684v1` (2020).

[3] Shental, N. *et al.* Efficient high-throughput SARS-CoV-2 testing to detect asymptomatic carriers. *Science Advances* **6** (2020).

[4] Sinnott-Armstrong, N., Klein, D. & Hickey, B. Evaluation of group testing for SARS-CoV-2 RNA. Preprint at `http://www.medrxiv.org/content/10.1101/2020.03.27.20043968v1` (2020).

[5] Baranyai, Z. On the factorization of the complete uniform hypergraph. In *Infinite and Finite Sets, Proc. Coll. Keszthely, 1973*, 91–108 (1975).

[6] Beth, T. Algebraische auflösungsalgorithmen für einige unendliche familien von 3–designs. *Le Matematiche* **29**, 105–135 (1974).

[7] Beth, T. *On resolutions of Steiner systems.* Arbeitsberichte des Instituts für Mathematische Maschinen und Datenverarbeitung, Informatik (Inst. für Math. Maschinen u. Datenverarbeitung (Informatik), Friedrich-Alexander-Univ. Erlangen-Nürnberg, 1978).

[8] Beth, T., Jungnickel, D. & Lenz, H. *Design Theory: Volume 1* (Cambridge University Press, Cambridge, 1999).

[9] Beth, T., Jungnickel, D. & Lenz, H. *Design Theory: Volume 2* (Cambridge University Press, Cambridge, 1999).

[10] D'yachkov, A. G., Vorobyev, I. V., Polyanskii, N. A. & Shchukin, V. Y. On a hypergraph approach to multistage group testing problems. In *2016 IEEE International Symposium on Information Theory (ISIT)*, 1183–1191 (IEEE, 2016).

[11] Tamm, U. Applications of Baranyai's theorem in information theory. In *Proceedings of 6th Benelux – Japan Workshop on Coding and Information Theory, Essen, 1996* (1996).

[12] Colbourn, C. J. & Dinitz, J. H. *Handbook of Combinatorial Designs* (Chapman and Hall/CRC, Boca Raton, FL, 2006), 2nd edn.

[13] Kautz, W. & Singleton, R. Nonrandom binary superimposed codes. *IEEE Transactions on Information Theory* **10**, 363–377 (1964).

[14] Reed, I. S. & Solomon, G. Polynomial codes over certain finite fields. *Journal of the Society for Industrial and Applied Mathematics* **8**, 300–304 (1960).

[15] Inan, H. A., Kairouz, P., Wootters, M. & Ozgur, A. On the optimality of the Kautz-Singleton construction in probabilistic group testing. *IEEE Transactions on Information Theory* **65**, 5592–5603 (2019).

[16] Dawson, D. A. & Sankoff, D. An inequality for probabilities. *Proceedings of the American Mathematical Society* **18**, 504–507 (1967).

[17] Galambos, J. Bonferroni inequalities. *Annals of Probability* **5**, 577–581 (1977).

[18] Finucan, H. M. The blood testing problem. *Journal of the Royal Statistical Society: Series C (Applied Statistics)* **13**, 43–50 (1964).

[19] Mutesa, L. *et al.* A pooled testing strategy for identifying SARS-CoV-2 at low prevalence. *Nature* **589**, 276–280 (2021).

[20] Berger, T. & Levenshtein, V. I. Asymptotic efficiency of two-stage disjunctive testing. *IEEE Transactions on Information Theory* **48**, 1741–1749 (2002).

[21] Levenshtein, V. I. A universal bound for a covering in regular posets and its application to pool testing. *Discrete Mathematics* **266**, 293–309 (2003).

[22] Bonis, A. D., Gasieniec, L. & Vaccaro, U. Optimal two-stage algorithms for group testing problems. *SIAM Journal on Computing* **34**, 1253–1270 (2005).

[23] Mezard, M. & Toninelli, C. Group testing with random pools: optimal two-stage algorithms. Preprint at `http://arxiv.org/abs/0706.3104v1` (2007).

[24] Mézard, M. & Toninelli, C. Group testing with random pools: Optimal two-stage algorithms. *IEEE Transactions on Information Theory* **57**, 1736–1745 (2011).

[25] Damaschke, P. & Muhammad, A. S. Randomized group testing both query-optimal and minimal adaptive. In *International Conference on Current Trends in Theory and Practice of Computer Science*, 214–225 (Springer, 2012).

[26] Coja-Oghlan, A., Gebhard, O., Hahn-Klimroth, M. & Loick, P. Optimal group testing. In *Proceedings of Thirty Third Conference on Learning Theory*, vol. 125, 1374–1388 (PMLR, 2020).

[27] Gebhard, O., Johnson, O., Loick, P. & Rolvien, M. Improved bounds for noisy group testing with constant tests per item. Preprint at `http://arxiv.org/abs/2007.01376` (2020).

[28] Scarlett, J. An efficient algorithm for capacity-approaching noisy adaptive group testing. In *2019 IEEE International Symposium on Information Theory (ISIT)*, 2679–2683 (IEEE, 2019).

[29] Dyachkov, A. G. & Rykov, V. V. A survey of superimposed code theory. *Problems of Control and Information Theory* **12**, 1–13 (1983).

[30] Erdös, P., Frankl, P. & Füredi, Z. Families of finite sets in which no set is covered by the union of r others. *Israel Journal of Mathematics* **51**, 79–89 (1985).

[31] Bilder, C. R. Group testing for identification. In *Wiley StatsRef: Statistics Reference Online* (American Cancer Society, 2019).

[32] Aldridge, M. Conservative two-stage group testing. Preprint at `http://arxiv.org/abs/2005.06617v1` (2020).

[33] Aldridge, M. Rates of adaptive group testing in the linear regime. In *2019 IEEE International Symposium on Information Theory (ISIT)* (IEEE, 2019).

[34] Aldridge, M., Johnson, O. & Scarlett, J. Group testing: an information theory perspective. Preprint at `http://arxiv.org/abs/1902.06002v2` (2019).

[35] Aldridge, M., Baldassini, L. & Johnson, O. Group testing algorithms: Bounds and simulations. *IEEE Transactions on Information Theory* **60**, 3671–3687 (2014).
