# Supplementary material for: Group testing via hypergraph factorization applied to COVID-19
Source: Nat Commun. 2022 Apr 5;13:1837. doi: 10.1038/s41467-022-29389-z (PMC8983763; doi:10.1038/s41467-022-29389-z)
Supplement: Supplementary file 2 — Reporting Summary [file 41467_2022_29389_MOESM2_ESM.pdf]

nature research

Corresponding author(s): Edgar Dobriban
Brian Cleary
Xihong Lin

Last updated by author(s): Mar 10, 2022

Reporting Summary

Nature Research wishes to improve the reproducibility of the work that we publish. This form provides structure for consistency and transparency in reporting. For further information on Nature Research policies, see our Editorial Policies and the Editorial Policy Checklist.

Statistics

For all statistical analyses, confirm that the following items are present in the figure legend, table legend, main text, or Methods section.

n/a	Confirmed	
☐	☒	The exact sample size (n) for each experimental group/condition, given as a discrete number and unit of measurement
☐	☒	A statement on whether measurements were taken from distinct samples or whether the same sample was measured repeatedly
☒	☐	The statistical test(s) used AND whether they are one- or two-sided<br>*Only common tests should be described solely by name; describe more complex techniques in the Methods section.*
☒	☐	A description of all covariates tested
☒	☐	A description of any assumptions or corrections, such as tests of normality and adjustment for multiple comparisons
☐	☒	A full description of the statistical parameters including central tendency (e.g. means) or other basic estimates (e.g. regression coefficient) AND variation (e.g. standard deviation) or associated estimates of uncertainty (e.g. confidence intervals)
☒	☐	For null hypothesis testing, the test statistic (e.g. F, t, r) with confidence intervals, effect sizes, degrees of freedom and P value noted<br>*Give P values as exact values whenever suitable.*
☒	☐	For Bayesian analysis, information on the choice of priors and Markov chain Monte Carlo settings
☒	☐	For hierarchical and complex designs, identification of the appropriate level for tests and full reporting of outcomes
☒	☐	Estimates of effect sizes (e.g. Cohen's d, Pearson's r), indicating how they were calculated

Our web collection on statistics for biologists contains articles on many of the points above.

Software and code

Policy information about availability of computer code

Data collection	A simulated population for the COVID-19 model capturing individual viral kinetics and epidemic dynamics was generated using previously published code available at https://github.com/cleary-lab/covid19-group-tests.
Data analysis	The efficiency and sensitivity of group testing methods were evaluated via simulation on a general statistical model as well as the above simulated population. Code is provided at https://github.com/dahong67/hyper-group-testing.

For manuscripts utilizing custom algorithms or software that are central to the research but not yet described in published literature, software must be made available to editors and reviewers. We strongly encourage code deposition in a community repository (e.g. GitHub). See the Nature Research guidelines for submitting code & software for further information.

Data

Policy information about availability of data

All manuscripts must include a data availability statement. This statement should provide the following information, where applicable:
- Accession codes, unique identifiers, or web links for publicly available datasets
- A list of figures that have associated raw data
- A description of any restrictions on data availability

No raw data were collected in this study. The data and analyses generated in this study are available at https://github.com/dahong67/hyper-group-testing and can be regenerated using the accompanying code. The simulated population for the COVID-19 model was obtained from previously published code available at https://github.com/cleary-lab/covid19-group-tests.

Field-specific reporting

Please select the one below that is the best fit for your research. If you are not sure, read the appropriate sections before making your selection.

- [x] Life sciences
- [ ] Behavioural & social sciences
- [ ] Ecological, evolutionary & environmental sciences

For a reference copy of the document with all sections, see nature.com/documents/nr-reporting-summary-flat.pdf

Life sciences study design

All studies must disclose on these points even when the disclosure is negative.

Sample size	All data are simulated, with sample sizes and replications described where relevant. A large array of pooling designs, including varying numbers of samples and numbers of pools, were evaluated. The upper limit of sample sizes was chosen based on an approximate number of samples that might be processed in a single facility on a daily basis in resource limited settings.
Data exclusions	No data were excluded.
Replication	Simulations were run with repeated random trials (up to 200,000 independent trials on each day of the epidemic) to assess variability in results.
Randomization	Simulated samples were chosen at random from a pool of 12,500,000 simulated individuals in each trial, with sample sizes specified where relevant. This study does not divide samples into experimental groups.
Blinding	Results in simulation were blinded. This study does not divide samples into experimental groups.

Reporting for specific materials, systems and methods

We require information from authors about some types of materials, experimental systems and methods used in many studies. Here, indicate whether each material, system or method listed is relevant to your study. If you are not sure if a list item applies to your research, read the appropriate section before selecting a response.

Materials & experimental systems

n/a	Involved in the study	
[x]	[ ]	Antibodies
[x]	[ ]	Eukaryotic cell lines
[x]	[ ]	Palaeontology and archaeology
[x]	[ ]	Animals and other organisms
[x]	[ ]	Human research participants
[x]	[ ]	Clinical data
[x]	[ ]	Dual use research of concern

Methods

n/a	Involved in the study	
[x]	[ ]	ChIP-seq
[x]	[ ]	Flow cytometry
[x]	[ ]	MRI-based neuroimaging
